# Distinct regulation of Tau Monomer and aggregate uptake and intracellular accumulation in human neurons

**DOI:** 10.1186/s13024-024-00786-w

**Published:** 2024-12-31

**Authors:** Amir T. Marvian, Tabea Strauss, Qilin Tang, Benjamin J. Tuck, Sophie Keeling, Daniel Rüdiger, Negar Mirzazadeh Dizaji, Hossein Mohammad-Beigi, Brigitte Nuscher, Pijush Chakraborty, Duncan S. Sutherland, William A. McEwan, Thomas Köglsperger, Stefan Zahler, Markus Zweckstetter, Stefan F. Lichtenthaler, Wolfgang Wurst, Sigrid Schwarz, Günter Höglinger

**Affiliations:** 1 Department of Neurology, School of Medicine, Klinikum rechts der Isar, Technical University of Munich, Munich, Germany; 2 German Center for Neurodegenerative Diseases (LMU), Klinikum, Germany; 3 Department of Neurology, University Hospital, Ludwig-Maximilians-Universität (LMU), Munich, Germany; 4 Munich Cluster for Systems Neurology (SyNergy), Munich, Germany; 5 UK Dementia Research Institute at the University of Cambridge, Cambridge, UK; 6 Department of Clinical Neurosciences, University of Cambridge, Cambridge, UK; 7 Department of Pharmacy, Ludwig-Maximilians-University of Munich, Munich, Germany; 8 Faculty for Chemistry and Pharmacy, Ludwig-Maximilians-Universität München, Butenandtstr. 5–13, 81377 Munich, Germany; 9 Department of Biotechnology and Biomedicine, Technical University of Denmark, DK-2800 Kgs., Lyngby, Denmark; 10 Interdisciplinary Nanoscience Centre (iNANO), Aarhus University, 8000, Aarhus C, Denmark; 11 Division of Metabolic Biochemistry, Biomedical Center (BMC), Ludwig-Maximilians-Universität München, Munich, Germany; 12 Department for NMR-based Structural Biology, Max Planck Institute for Multidisciplinary Sciences, Am Fassberg 11, 37077 Gӧttingen, Germany; 13 Department of Translational Brain Research, DZNE-German Center for Neurodegenerative Diseases, 81377 Munich, Germany; 14 German Center for Neurodegenerative Diseases (DZNE), Von-Siebold-Str. 3a, 37075 Gӧttingen, Germany; 15 Neuroproteomics, School of Medicine, Klinikum rechts der Isar, Technical University of Munich, Munich, Germany; 16 Institute of Developmental Genetics, Helmholtz Zentrum München, Neuherberg, Germany; 17 School of Life Sciences, Technical University Munich, Freising, Germany; 18 Haag, Geriatric Clinic Haag, Oberbayern, Germany; 19 Department of Neurology, Hanover Medical School, Hanover, Germany; 20 Center for Systems Neuroscience, Hanover, Germany

**Keywords:** Neurodegeneration, Cell-to-cell spreading, Extracellular Tau, Uptake, HSPGs, LRP1, VPS35

## Abstract

**Background:**

The prion-like spreading of Tau pathology is the leading cause of disease progression in various tauopathies. A critical step in propagating pathologic Tau in the brain is the transport from the extracellular environment and accumulation inside naïve neurons. Current research indicates that human neurons internalize both the physiological extracellular Tau (eTau) monomers and the pathological eTau aggregates. However, similarities or differences in neuronal transport mechanisms between Tau species remain elusive.

**Method:**

Monomers, oligomers, and fibrils of recombinant 2N4R Tau were produced and characterized by biochemical and biophysical methods. A neuronal eTau uptake and accumulation assay was developed for human induced pluripotent stem cell-derived neurons (iPSCNs) and Lund human mesencephalic cells (LUHMES)-derived neurons. Mechanisms of uptake and cellular accumulation of eTau species were studied by using small molecule inhibitors of endocytic mechanisms and siRNAs targeting Tau uptake mediators.

**Results:**

Extracellular Tau aggregates accumulated more than monomers in human neurons, mainly due to the higher efficiency of small fibrillar and soluble oligomeric aggregates in intraneuronal accumulation. A competition assay revealed a distinction in the neuronal accumulation between physiological eTau Monomers and pathology-relevant aggregates, suggesting differential transport mechanisms. Blocking heparan sulfate proteoglycans (HSPGs) with heparin only inhibited the accumulation of eTau aggregates, whereas monomers’ uptake remained unaltered. At the molecular level, the downregulation of genes involved in HSPG synthesis exclusively blocked neuronal accumulation of eTau aggregates but not monomers, suggesting its role in the transport of pathologic Tau. Moreover, the knockdown of LRP1, as a receptor of Tau, mainly reduced the accumulation of monomeric form, confirming its involvement in Tau’s physiological transport.

**Conclusion:**

These data propose that despite the similarity in the cellular mechanism, the uptake and accumulation of eTau Monomers and aggregates in human neurons are regulated by different molecular mediators. Thus, they address the possibility of targeting the pathological spreading of Tau aggregates without disturbing the probable physiological or non-pathogenic transport of Tau Monomers.

**Supplementary Information:**

The online version contains supplementary material available at 10.1186/s13024-024-00786-w.

## Background

Abnormal aggregates of microtubule-associated protein Tau have been found in the brains of patients with a spectrum of neurodegenerative diseases called tauopathies [1–3]. Immunocytochemical analysis of postmortem brain tissue of patients with tauopathies revealed a spatiotemporal correlation between the disease progression and the spreading of abnormal Tau inclusions [4–8]. A growing body of evidence suggests that cell-to-cell transfer of pathogenic Tau seeds promotes the abnormal aggregation of physiological monomeric Tau, leading to the exponential amplification of Tau pathology spreading through the brain [9]. A prion-like propagation theory consisting of three main steps has been suggested as a mechanism for this phenomenon. First, pathogenic seeds are secreted from the diseased cells into the extracellular environment. Second, healthy cells take up the extracellular seeds and accumulate them inside the cytosol if not expelled or digested. Finally, the internalized pathogenic seeds induce aggregation of normal physiological Tau, producing more seeds to be released for initiating the next cycle [10].

Despite numerous attempts over recent decades, the nature of seed-competent Tau species has remained elusive [11]. Various aggregated species form during the process of fibril formation, including intermediate aggregates like multimers, oligomers, and small fibrils that finally lead to the formation of large fibrils [12]. Likewise, various species of Tau have been identified in postmortem tissue, cerebrospinal fluid (CSF), and interstitial fluid (ISF) in tauopathies, including monomeric, oligomeric, small and large fibrillar forms [1, 13]. Thus, evaluating features, including efficient neuronal uptake, cytosol entry, and seeding endogenous aggregation, is critical for employing any fabricated seed to study spreading in vitro.

During the last decade, remarkable progress has been achieved in understanding the molecular mechanism of Tau uptake as a critical step in pathology spreading [14]. The process of endocytosis for both monomeric and aggregated Tau was found to be temperature-dependent active transports [15]. Diverse cellular mechanisms have been reported for the cellular internalization of Tau species, including clathrin-mediated endocytosis (CME), clathrin-independent endocytosis (CIE), and macropinocytosis [14]. Heparan sulfate proteoglycans (HSPGs) have been identified as the first mediators of Tau uptake [16–18] as well as low-density lipoprotein receptor-related protein 1 (LRP1) [19]. So far, the role of these endocytic mechanisms and molecular mediators on various Tau species remains poorly understood.

The physiological functions of Tau protein have been mainly associated with intracellular activities [20]. However, under physiological conditions, Tau is also present in body fluids, especially brain fluids, e.g., CSF and ISF [21]. The enhanced secretion of Tau by neuronal activity [22–24] and the presence of specific membrane receptors for Tau suggests that the uptake of extracellular Tau (eTau) is a well-regulated physiological process [19]. Despite the lack of direct evidence, this raises the possibility of a physiological function for eTau [25]. Thus, it is essential to address whether the physiological transport of native Tau Monomers overlaps or is distinct from the cell-to-cell transport of pathological Tau aggregates for developing therapeutic strategies to target Tau spreading.

In this study, we first generated recombinant 2N4R Tau aggregates and compared their neuronal uptake with monomeric Tau. Both in iPSCNs and LUHMES neurons, we observed a higher intracellular accumulation of aggregates. The aggregated mixture was fractionated to obtain a homogenous population and characterized using biochemical and biophysical assays. Among these fractions, oligomeric and small fibrillar species were found to be competent seeds due to their high uptake efficiency, cytosol entry, and capacity to induce endogenous aggregation. Studying the uptake competition between eTau aggregates and monomers revealed a low interspecies competition, suggesting distinctions in transport mechanisms of pathological and physiological species. Further mechanistic studies indicated that LRP1 mainly regulates the uptake of Tau Monomers, and HSPGs mainly regulate the uptake of Tau aggregates, while other internal processes may be involved in this distinct accumulation. Our findings shed light on a novel opportunity for specific targeting of pathological Tau transport with a negligible impact on the physiological transport of eTau.

## Methods

### Production of recombinant tau protein

Tau protein was expressed and purified as described before [26] with some modifications. In brief, *Escherichia coli* BL21 (Rosetta 2 (DE3), Merck) were transformed by 2N4R Tau plasmid (pRK172) and cultured in terrific broth supplemented with ampicillin at 37 °C. Tau expression was induced at OD: 1–1.5 by the addition of 1 mM IPTG. After 6 h of incubation at 37 °C and 180 rpm, cells were harvested by centrifugation at 4000 g for 10 min at 4 °C. The pellet was resuspended in buffer A (20 mM MES pH 6.8, 50 mM NaCl, 1 mM MgSO4, 1 mM EGTA, 1 mM DTT, 1 mM PMSF) and froze at -20 °C overnight. After a freeze-thaw cycle, the suspension was sonicated 3 s/ml (3 s on, 3 s off) with 50% amplitude (UP200St, Hielscher). Streptomycin sulfate (MP Biomedicals) was added up to 1% and the lysate was clarified by centrifugation at 10,000 g for 10 min at 4 °C. The supernatant was collected and NaCl was added to reach 200 mM. Then the extract was boiled at 95 °C for 15 min and incubated on ice for 10 min. The precipitate was removed by centrifugation at 20,000 g for 10 min at 4 °C. The supernatant was collected and dialyzed in buffer A overnight with two times changes. Semi-purified protein loaded into SP HP HiTrap (GE Healthcare) and eluted with a 0–50% gradient of buffer B (20 mM MES pH 6.8, 500 mM NaCl, 1 mM MgSO4, 1 mM EGTA, 1 mM DTT, 1 mM PMSF) by using an ÄKTA™ pure (GE Life Sciences). The fractions were analyzed with Bis-Tris SDS-PAGE 12% and stained with Imperial protein stain (Thermo Fisher Scientific). The pooled fractions were concentrated using 10 kDa Vivaspin 15R (Sartorius) and run into a Superdex 200 Increase 10/300 GL column (GE Healthcare) pre-equilibrated in PBS. The protein was eluted at a 0.3 ml/min rate, and fractions were analyzed with absorbance at 215 nm. The concentration of pooled fractions was determined by the Bicinchoninic acid (BCA) assay kit (Thermo Fisher Scientific), and the recombinant protein was diluted to 6 mg/ml, aliquoted, and preserved at -80 °C following a snap-freezing step in liquid nitrogen.

### Fluorescence labeling of Tau Monomers and aggregates

For labeling of Tau Monomers, the recombinant protein at the concentration of 3 mg/ml (~ 65 µM) was labeled with ATTO488-NHS ester or ATTO633-NHS ester fluorescence dye (ATTO-TEC, Siegen, Germany) based on the manufacturer’s instruction. In brief, the protein solution was adjusted to pH 8.3 with a 0.2 M sodium bicarbonate solution (Sigma-Aldrich) and then incubated with 200 µM dye at room temperature for 1 h in the dark. The unbound dye was removed by Bio-Spin 6 size exclusion spin columns (Bio-Rad Laboratories).

For labeling of the Tau aggregates, two approaches were used: (A) The “pre-aggregation labeling” was performed by mixing 10% of pre-labeled monomers (~ 1 label per monomer) with 90% of unlabeled monomers before the incubation in the fibrillization condition. Although the pre-aggregation labeling ensures an equal degree of labeling between monomers and aggregates, this approach reduces the yield of fibril formation, perhaps due to the interference of labeling at residues involved in Tau fibrillization. Thus, a second approach was used to increase the fibril formation yield. (B) The “post-aggregation labeling” was performed by incubating the aggregated mixture after the fibrillization process with 200 µM ATTO dye, similar to the monomers labeling protocol. The excess unbound labels were removed by washing steps during the fractionation process. The labeling was verified by running the protein on SDS-PAGE followed by fluorescence imaging. The degree of labeling (DOL) was calculated by using the below equation:

$$\:\text{D}\text{O}\text{L}=\frac{{A}_{max}\times\:\text{M}\text{W}}{\left[\text{P}\text{r}\text{o}\text{t}\text{e}\text{i}\text{n}\right]\:\times\:\:{\epsilon\:}_{dye}}$$

Where $\:{A}_{max}$ is the absorbance of ATTO488 at 500 nm, MW is the molecular weight of 2N4R Tau (= 21.8), $\:{\epsilon\:}_{dye}$ is the extinction coefficient of the dye at its maximum absorbance (ATTO488 = 90,000). The protein concentration of Tau was measured by BCA assay.

The Guanidine Hydrochloride (GuHCl) unfolding assay was performed by treating 3 µg (~ 1 µl) Tau Monomers and aggregates with 2 M GuHCl for 5 min. Unlabeled Tau aggregates were used for ThT assay, and labeled Tau aggregates were used to study the change in ATTO488 fluorescence. The mixture was diluted in ~ 100 µl of ThT assay buffer or PBS before the fluorimetry.

### Fibril formation of recombinant tau protein

The fibril formation of 3 mg/ml 2N4R Tau (~ 65 µM) was induced by 130 µM Heparin (~ 3000 kDa, MP Biomedicals) in PBS buffer pH 7.4 supplemented with 1 mM DTT at 37 °C in a 2 ml microtube. Fibrillization of 100–200 µl mixture was accelerated with 1400 rpm shaking in the presence of a 3 mm glass bead using a Thermomixer (Eppendorf Thermomixer). The fibrillization process was monitored by sampling over time, followed by thioflavin T (ThT) fluorescence measurement. For ThT analysis, 2 µl of aggregation sample were added to 98 µl of 10 µM ThT in 10 mM Tris pH 8.0, and fluorescence measurement was done in black 96 well-plate at Ex: 488, Em: 520 by using a CLARIOStar microplate reader (BMG Labtech, Offenburg, Germany). The Finke-Watzky [27] equation was used to fit the normalized ThT data.

The co-factor-free aggregation of 2N4R tau was performed using the previously described protocol [28]. Briefly, 25 µM of protein were aggregated at 37 °C in 25 mM HEPES, 10 mM KCl, 5 mM MgCl_2_, 3 mM TCEP, 0.01% NaN_3_, pH 7.2 buffer in a 96 well plate using a Tecan spark plate reader. Three PTFE beads along with double orbital shaking, were used to promote the aggregation. Thioflavin-T (ThT) at a final concentration of 50 µM was used to monitor the aggregation kinetics.

### Fibril formation of bovine serum albumin (BSA)

The BSA fibrillization mixture was prepared by dissolving 5 mg/ml BSA in 20 mM Tris pH 7.4. 100–200 µl of the mixture was incubated at 70 °C with 1000 rpm shaking in a 2 ml microtube with a 3 mm glass bead using a Thermomixer (Eppendorf Thermomixer). For making fluorescent BSA aggregates, 4% BSA-CF488A (Biotium) was added to 96% unlabeled BSA before the incubation in the fibrillization condition. The amyloid fibril formation was confirmed by using PROTEOSTAT^®^ Protein aggregation assay (ENZ-51023, Enzo).

### Fractionation of tau aggregates

The labeled or unlabeled Tau aggregates obtained from the fibril formation process were divided into four fractions of large fibrils, small fibrils, soluble oligomers and monomers via a stepwise procedure as follows: (I) The fibrillization mixture was transferred into 1.5 ml microtubes with portions of 100–150 µl and centrifuged 16,000 g for 1 h at 4 °C. The pellet was dispersed and washed twice with 100 µl of PBS with the same centrifugation setting and finally dispersed in PBS as large fibrils (L-fib). (II) The supernatant of step one was collected and transferred into 1.5 ml Eppendorf ultracentrifuge tubes with portions of 100 µl and subjected to ultracentrifugation 100,000 g for 30 min at 4 °C. The pellet was washed with 100 µl PBS with the same centrifugation setting and finally was dispersed in PBS as small fibrils (S-fib). (III) The supernatant of the second step was collected and filtered through a 100 kDa ultrafilter (Amicon Ultra 0.5 ml, Millipore) with portions of 100–500 µl for 10 min at 4 °C. The retained phase was washed twice with 500 µl PBS and concentrated as soluble oligomers (Oligo). (IV) The pass-through of step three was washed twice with 500ul PBS using a 10 kDa ultrafilter (Amicon Ultra 0.5 ml, Millipore). Finally, it was concentrated as fibrillization-derived monomers (F-mono). The fractions were aliquoted and stored after a snap freeze in liquid nitrogen at -80 °C.

### Native state immunoblot

The samples were collected at different times during the fibrillization process or after fractionation to examine the conformational status of the aggregates using a dot-blot assay. Samples were diluted 1:15 in PBS and loaded into 0.2 μm nitrocellulose blotting membrane (GE, 10600004) using a dot blot chamber (11055, Life technologies). Membranes were washed three times with PBS, then released from the chamber and blocked for 1 h at RT in 30% Roti-Block solution (Carl Roth, Karlsruhe, Germany) before the overnight incubation with primary antibodies at 4 °C under continuous agitation. After 3 times washing in Tris-buffered saline (TBS) supplemented with 0.05% Tween-20 (Sigma-Aldrich) (TBST), HRP-coupled secondary antibodies were incubated for 1 h at room temperature. After another round of washing steps in TBST, for visualization, membranes were incubated in Clarity Western ECL Substrate (Bio-Rad Laboratories), and imaging was done with Odyssey Fc (LI-COR Biotechnology, Lincoln, NE) imaging system. The primary antibodies used in this study: Tau-5 (1:1000; MAB361, Merck Millipore, Billerica, MA), a general monoclonal anti-Tau antibody; TNT-1 (1:1000; MABN471, Merck Millipore, Billerica, MA) identifying the phosphatase-activating domain in the N-terminal region of Tau, which is only exposed in a pathological conformation [29]; TOMA-1 (1:500; MABN819, Merck Millipore, Billerica, MA) being an anti-Tau oligomer-specific conformational antibody [30]; MC1 (1:500; a gift from Dr. Peter Davis) indicating a pathological conformation by binding two discontinues AD-specific epitopes at N-terminal and microtubule-binding domain [31]. The anti-mouse IgG (1:2000; Vector Laboratories, Burlingame, CA) was used as a secondary antibody.

### Biophysical characterization of tau species

Size exclusion chromatography was performed by pre-equilibration of Superdex 200 Increase 10/300 GL (GE Healthcare) with two column volumes of elution buffer (PBS) followed by loading 500 µl of samples at 0.1 mg/ml concentration. Samples were injected into an ÄKTA™ pure (GE Life Sciences) and run at flow rates of 0.3 mL/minute. The elution profile was monitored at wavelengths of 214 and 280 nm. The fractions were collected and further analyzed using dot-blot assay and dynamic light scattering (DLS). DLS measurements were carried out using a Malvern Zetasizer-Nano instrument with a 4 mW He-Ne laser (633 nm) in a water suspension at 0.03 mg/ml concentration.

Atomic force microscopy (AFM) imaging was performed using the NanoWizard^®^ 4 (JPK, Berlin, Germany) and SPM software with an integrated Axiovert 200 inverted microscope (Zeiss, Jena, Germany). The cantilevers qp-BioAC-CB1 (NanoWorld, Neuenburg, Switzerland) with a resonance frequency of 90 kHz and a spring constant of 0.3 N/m were used and calibrated with the contact-free method. The QI™ Mode (Advanced Imaging) and the following parameter settings were used for the image acquisition: setpoint 0.4–0.6 nN; z-length 86–126 nm and pixel time 2.2–5.5 ms. The sample was diluted in distilled water and dried on a freshly prepared surface of the highest grade V1 AFM Mica Discs, 10 mm (Ted Pella). Mica discs were washed three times with distilled water. The measurements were performed in air at ambient temperature. All images were processed, optimized, and zoomed in with the data processing software version 6.0.50 (JPK, Berlin, Germany). The first step was subtracting a polynomial fit from each scan line independently. A histogram was calculated for each scan line, and the data between the lower (0%) and upper (70%) limits was used to fit the polynomial. The next step was replacing outlier pixels with the median value of neighboring pixels. Lastly, a low-pass filter was applied (2-dimensional Savitzky–Golay smoothing; smoothing width: 5, order: 4).

Transmission electron microscopy (TEM) of Tau aggregates was performed as described before [32]. In brief, 3 µl of Tau aggregates were loaded onto glow-discharged 400 mesh Formvar/carbon grids (EM resolutions) for 20 s, blot-dried, and stained three times with uranyl formate (3 µl, 15 s for each time). TEM imaging was carried out by using a Tecnai G2 Spirit BioTWIN (FEI) operating at 120 kV acceleration. Images were obtained on a TemCam-F416(R) (TVIPS) CMOS camera.

UV-Circular dichroism (UV-CD) spectroscopy of different fractions of Tau aggregates was performed via a Chirascan V100 CD spectrometer (Applied Photophysics) by loading 2–6 µg of protein samples. The UV-CD spectra were recorded between 190 and 250 nm with a step size of 1 nm and a scanning speed of 10 nm/min using a 1-mm path-length cuvette at room temperature.

Gradient centrifugation of iodixanol was performed by manual filling of 10 ml gradient columns with ten portions of 1 ml OptiPrep of 5–50% (Sigma, D1556) in 14 ml centrifuge tubes (Beckmann Coulter, 344060). A 500 µl of each fraction of ATTO-488 labeled Tau, including L-fib, S-fib, and soluble fraction (the supernatant of 30 min centrifugation at 100.000 g at 4 °C), were loaded on top, and then columns were subjected to ultracentrifugation 250,000 g at 4 °C for 3 h. The 10 ml columns were fractionated manually into 40 fractions of 250 µl in black 96-well plates, and the fluorescence of each fraction was measured at Ex: 488, Em: 535 by using a CLARIOStar microplate reader (BMG Labtech, Offenburg, Germany).

### Induced pluripotent stem cells derived neurons (iPSCNs) culture and differentiation

For the ease of differentiation via lentiviral transduction, small molecule neuroprogenitor cells (smNPCs) were generated from induced pluripotent stem cells via embryoid body formation and stable integration of an inducible *NGN2* vector, as described before [33, 34]. *NGN2*_smNPCs were cultured in N2B27 medium (48.425% DMEM/F12 Medium, 48% Neurobasal Medium, 0.5% N2-supplement, 1% B27 supplement without Vitamin A (Life Technologies, Carlsbad, CA, United States), 0.025% Insulin (Sigma-Aldrich, St. Louis, MO, United States), 0.5% Non-essential amino acids, 0.5% GlutaMax, 1% Penicillin/Streptavidin (Life Technologies, Carlsbad, CA, United States) and 0.05% β-Mercaptoethanol) supplemented with 0.5 µM Purmorphamine, 3 µM CHIR99021 and 64 µg/ml ascorbic acid (Th. Geyer, Renningen, Germany). During expansion and differentiation, cells were maintained at 37 ‌°C with 5% CO2 in a tissue culture incubator.

For the neuronal differentiation, plates were coated by overnight incubation at 4 °C with 100 µg/ml Poly-L-ornithine (PLO) (Sigma-Aldrich, St. Louis, MO, United States) diluted in 0.1 M borate buffer at pH 8.4 and subsequent incubation with 10 µg/ml Laminin (Sigma-Aldrich, St. Louis, MO, United States) at 37 °C. NGN2_smNPCs were directly seeded in an induction medium containing N2B27 medium supplemented with 2.5 µg/ml doxycycline (Sigma-Aldrich, St. Louis, MO, United States) and 2 µM DAPT (Cayman, Ellsworth, United States). The medium was entirely changed at day 3 to N2B27 supplemented with 2.5 µg/ml doxycycline, 10 µM DAPT, 10 ng/ml BDNF, 10 ng/ml GDNF, 10 ng/ml NT 3 (PeproTech, Princeton, NJ, United States) and 0.5 µg/ml laminin. From day 6, only 50% of the medium was changed every 3 days with fresh differentiation medium without doxycycline and DAPT.

### Lund human mesencephalic (LUHMES) cell culture and differentiation

As described before [35], LUHMES were cultured in flasks (EasYFlasks, Nunclon DELTA, VWR, Darmstadt, Germany) coated with 50 µg/ml PLO (Sigma-Aldrich, St. Louis, MO) in expanding medium containing DMEM-F12 (Sigma-Aldrich) supplemented with 1% N2 supplement (Life Technologies, Carlsbad, CA) and 0.04 µg/ml basic fibroblast growth factor (bFGF; PeproTech, Rocky Hill, CT). For differentiation, plates were coated first with 50 µg/ml PLO (Sigma-Aldrich) and then with 5 µg/ml bovine fibronectin (Sigma-Aldrich). Cells were seeded directly in the differentiation medium containing DMEM/F12 with 1% N2 supplement, 1 µg/ml tetracycline, 0.5 µg/ml dibutyryl cyclic-AMP (Sigma-Aldrich), and 2 ng/ml glial cell-derived neurotrophic factor (GDNF; R&D Systems, Minneapolis, MN). During expansion and differentiation, cells were maintained at 37 °C with 5% CO2 in a tissue culture incubator.

### Cell viability assay

For studying the toxicity of Tau species, smNPCs were cultures for differentiation in transparent bottom black 96 well-plates (PerkinElmer) and, at day 13–15th of differentiation, treated with 250 nM (Monomer equivalent) of Tau species for 24 h. Then media was removed and viability was assessed using the cell viability indicator of the neural outgrowth staining kit based on the company instruction (A15001, life technologies). The fluorescence was measured by CLARIOStar microplate reader (BMG Labtech, Offenburg, Germany) with a matrix of 15 × 15 from the bottom (Ex: 480 nm, Em: 520–535 nm).

### Tau endogenous aggregation biosensor assay

Seeding in HEK293T overexpressing mutant P301S 0N4R Tau, C-terminally tagged with Venus protein, was performed as described before [36]. In brief, cells were cultured in complete DMEM (C-DMEM) with 10% (vol/vol) FCS, 100 U/ml penicillin, and 100 µg/ml streptomycin at 37 °C in a 5% CO2 atmosphere. Cells were cultured on poly-L-lysine (Sigma, P4707) coated transparent bottom black 96-well plates in C-DMEM (PERK6055302, PerkinElmer) for seeding. On day 2, the media were discarded and the wells were washed twice with PBS. Seeding was induced by adding OptiMEM (Gibco™, 51985026) containing 1% (vol/vol) Lipofectamine 2000 (Life Technologies) and 400 nM (Monomer equivalent) of Tau species for 1 h. Next, the seeding medium was aspirated and C-DMEM was replaced. On day 4, the media were changed to FLuoroBrite DMEM (Gibco™, A1896701) containing 1X backdrop suppressor (Thermo Fischer Scientific, B10512), and cells were imaged by fluorescence microscope and plate reader.

### Neuronal entry assay

A live-cell neuronal entry assay was carried out, as described before [37], by using a split luciferase called NanoLuc (Nluc) composed of a large 18 kDa subunit (LgBiT) and a small 11 amino acid peptide (HiBit) forms a complementation reporter [38]. HiBiT-tagged Tau was added to the extracellular medium of primary mouse neurons expressing LgBiT. Tuck et al. previously showed that human and mouse neurons had similar Tau uptake dependencies [37]. To do this, using the abovementioned method, 0N4R P301S-Tau-HiBiT were fibrillized and fractionated into four different fractions of L-fib, S-fib, Oligo, and F-mono. Primary neurons were prepared from postnatal day 0/1 C57BL/6 mouse pups and infected at 2 days in vitro (DIV) with AAV1/2 hSyn::-eGFP-P2A-LgBiT-nls particles at a multiplicity of 50,000 genome copies per cell. On DIV 7, 2 ug/ml of each Tau-HiBiT species was prepared in maintenance media. 50% of the media was used for an in vitro reconstitution assay, and the remainder was used for neuronal entry assays. For in vitro reconstitution, the total signal in the maintenance media (RLU in media) was quantified by the addition of excess recombinant LgBiT for 30 min. With the remaining media, neurons were 100% media changed and incubated with Tau-HiBiT preparations for the depicted amount of time. Cytosolic entry was quantified (RLU in cells), followed by incubation for 42 min with PrestoBlue cell viability reagent according to manufacturer instructions (Thermo Fisher Scientific). Fluorescence intensity was quantified (excitation 540–570 nm; emission 580–610 nm) using the CLARIOstar microplate reader (BMG Labtech). Total viable cells per well were calculated using a standard curve of viable cells per well and adjusted for background fluorescence. Percent cytosolic entry normalized to cells was calculated by dividing the RLU in cells by RLU in media and normalizing it to total viable cells per well.

### Uptake and accumulation assay

smNPCs or LUHMES were seeded in a black clear-bottom 96-well tissue culture plate (PERK6055302, PerkinElmer) for differentiation. Neurons were treated with fluorescently labeled Tau species in differentiation media after complete media removal. The concentration and incubation time varied for different experiments and were specified in the result section. The FL-Tau-containing media were removed and 100 µl FluoroBrite DMEM (Gibco™, A1896701) containing 1X BackDrop background suppressor (B10512, Thermo Fisher Scientific) was added. The fluorescence of ATTO488 was scanned by excitation at 490 nm and emission at 510–530 nm (Focal length: 0.9 mm, Gain: 2200) with a matrix of 15 × 15 from the bottom of the wells by using a CLARIOStar microplate reader (BMG Labtech, Offenburg, Germany). The fluorescence intensity of each well was normalized by dividing to the untreated cells as a blank for background fluorescence. The representative fluorescence images were taken by live imaging using Leica DMI6000 B (Leica Microsystems, Germany). To validate the comparability of cellular uptake and accumulation under various treatments and conditions, cells’ viability was examined using the 0.1 μm Calcein-AM (Thermo, C3100MP) treatment for 30 min, and measurement was performed in the presence of 1X BackDrop background suppressor (B10512, Thermo Fisher Scientific). The treatment conditions were adjusted in the none-toxic range.

Recombinant 2N4R Tau Monomers were labeled with ATTO488 to compare the uptake of monomer and aggregated mixtures. Then, the labeled monomers were mixed 1:9 with unlabeled monomers. Next, the mixture was divided into two parts; part A was kept at 4 °C, and part B was incubated in the fibrillization condition described as “pre-aggregation labeling” in the fibril formation section. iPSCNs at day 13–18 of differentiation were treated with part A (Mono) and part B (Agg) at various concentrations and different incubation times (at 37 °C) to compare the kinetics and titration. For comparing the uptake of Tau fractions, “post-aggregation labeling” was used as described in the fibril formation section. iPSCNs at day 13–18 of differentiation and LUHMES at day 6–8 of differentiation were treated with labeled fractions at various concentrations and incubation times to compare the kinetics and titration.

For the Tau competition assay, cells treated with 50 or 100 nM (Monomer equivalent) of ATTO488 labeled Tau Mono or aggregates and at the same time with 4- or 5-times higher concentrations of unlabeled species for 16–20 h. Since the uptake of Mono was lower than aggregates, a 10-fold higher degree of labeling (labeling efficiency around 2 compared to ~ 0.1–0.2 for aggregates) was used. It is crucial that competing aggregates are from the same batch of aggregate preparation.

To study the impact of the small molecules on Tau uptake, iPSCNs were treated at days 13–18 of differentiation. For the small molecules in Table 1A, cells were treated for 30 min at a specified concentration. After a washing step (with 100 ul PBS), they were incubated with 250 nM Tau for 3 h. For the small molecules in Table 1B, cells were co-treated with small molecules at a specified concentration and Tau at 25–50 nM for 18–20 h. For viability assessments, cells were treated with a standard medium for 3 h and then incubated for 30 min with 0.1 μm Calcein-AM (Thermo, C3100MP).

**Table 1:** List of small molecules used for studying the intracellular accumulation of eTau

Name	Company	Catalog
A		
Chlorpromazine	Santa Cruz	sc-357,313
Cytochalasin D	MP Biomedicals	0215077101
5-(N-Ethyl-N-isopropyl) -Amiloride (EIPA)	Santa Cruz	sc-202,458
Dyngo-4a	selleckchem	S7163
Genistein	Santa Cruz	sc-3515
Nystatin	Sigma	N6261
B		
bafilomycin A1	Santa Cruz	sc-201,550 A
Chloroquine diphosphate salt	Sigma	C6628
MG132	Tocris Bioscience	1748
Atropine Sulfate	Sigma	PHR1379
Pirenzepine Dihydrochloride	Sigma	15,378,983
Heparin sodium salt ~ 3000 kDa	MP Biomedicals	19,411,480

For the pre-treatment experiments, LUHMES were treated with 100 µM Heparin, 200 nM monomers, or small fibrils (Monomer equivalent) for two hours on day six. Then, the media was removed, and cells were washed once with PBS before treatment with fluorescently labeled Tau Monomers or small fibrils.

For the gene-knockdown experiments, cells were treated with 10 nM siRNAs (LRP1, EXT1, and EXT2: siPOOLs from siTOOLs, VPS35: Silencer Select siRNAs from Thermo Fischer) in the presence of 0.075 µl/well RNAiMax lipofectamine (Thermo Fisher Scientific, 13778075) in 1:1 differentiation medium to OptiMEM (Gibco™, 51985026) for 24 h. siRNA treatments were performed on day 2 post-seeding, and the media was changed the next day. iPSCNs and LUHMES neurons were treated with 25–50 nM (Monomer equivalent) of Tau Monomers and small fibrils for 18–20 h on days 10–12 and 6–8 of differentiation, respectively. The knockdown was confirmed by immunoassay.

### Western blot

iPSC-derived neurons and LUHMES neurons were differentiated in 6-well plates and treated with siRNA as described before. The whole cell extract was collected by radioimmunoprecipitation assay (RIPA) lysis buffer (Thermo, 89901) as described by the manufacturer. For western blot, 20–40 ug of cell extracts were loaded in 4–12% Bolt Bis-Tris precast Gels (Invitrogen, NW04127BOX) and were run for 20 min by a PowerEase Touch 350 W (Invitrogen, PSC350M), then were transferred to methanol-activated low fluorescence 0.2 μm PVDF membrane (GE, 10600022) by a PowerBlotter XL (Invitrogen, PB0013) at 25 V, 2.5 A for 13 min. The membrane was blocked with 1X Roti-Block (Carl Roth, A151.2) for 1 h at RT and incubated overnight at 4 °C with primary antibodies in 1X Roti-Block diluted in wash buffer (0.05% Tween-20 in Tris-buffer saline (TBS-T) pH 7.5). Following 3 times washing in TBS-T, the membranes were incubated with a secondary antibody in 1X Roti-block in TBS-T for 2 h at RT. After three times rinsing with TBS-T, the membranes were incubated 5 min at RT with SuperSignal West Pico PLUS Chemiluminescent Substrate (Thermo, 34580) and imaging was performed by iBright CL1500 imaging system (Invitrogen, A44114). Loading control was performed by incubation of the membranes with a β-actin (1:1000, Cell Signalling Technology 13E5) and the following primary antibodies were used in this study: LRP1 (1:1000; Abcam ab92544), EXT2 (1:100, Santa Cruz Biotechnology sc514092), VPS35 (1:1000, Cell Signalling Technology E6S4I). The following secondary antibodies were used: HRP-coupled anti-mouse (1:2000; Cell Signalling Technology 7076), or -rabbit antibody (1:5000; Cell Signalling Technology 7074).

### Confocal imaging

smNPCs and LUHMES were plated on 8-well ibidi µ-slides (ibidi, Gräfelfing, Germany) and incubated in the differentiation medium for 15 and 7 days, respectively. Following the differentiation, neurons were treated with Tau Mono labeled with ATTO633 and Tau S-fib labeled with ATTO488 for 3 h. Imaging was done immediately or 21 h after treatment. In the latter case, cells were washed and incubated in the differentiation medium until imaging. Before imaging, cells were stained with 1 µM Cell Trace™ Calcein Red-Orange (Thermo Fisher Scientific) for 30 min. Then the media were changed to FluoroBrite DMEM containing 1X Backdrop™ background suppressor (Thermo Fisher Scientific). Live Z-stack images were taken using Zeiss LSM 880 (Carl Zeiss, Oberkochen, Germany) via a 63x oil immersion objective and 2X digital zoom.

### Statistical analysis

Statistical analysis was performed using GraphPad Prism 8.0.2 (GraphPad Software, La Jolla, CA, USA) or the Excel data analysis package. All data shown in the figures are presented as mean ± standard error of the mean (SEM) or standard deviation (SD). All data were analyzed via one-way ANOVA followed by Dunnett’s post hoc test, except for the siRNA experiment, where two-way ANOVA followed by the Sidak test was performed. P-values < 0.05 were considered statistically significant.

## Results

### Generation and characterization of heparin-induced recombinant tau aggregates

**Fig. 1:**
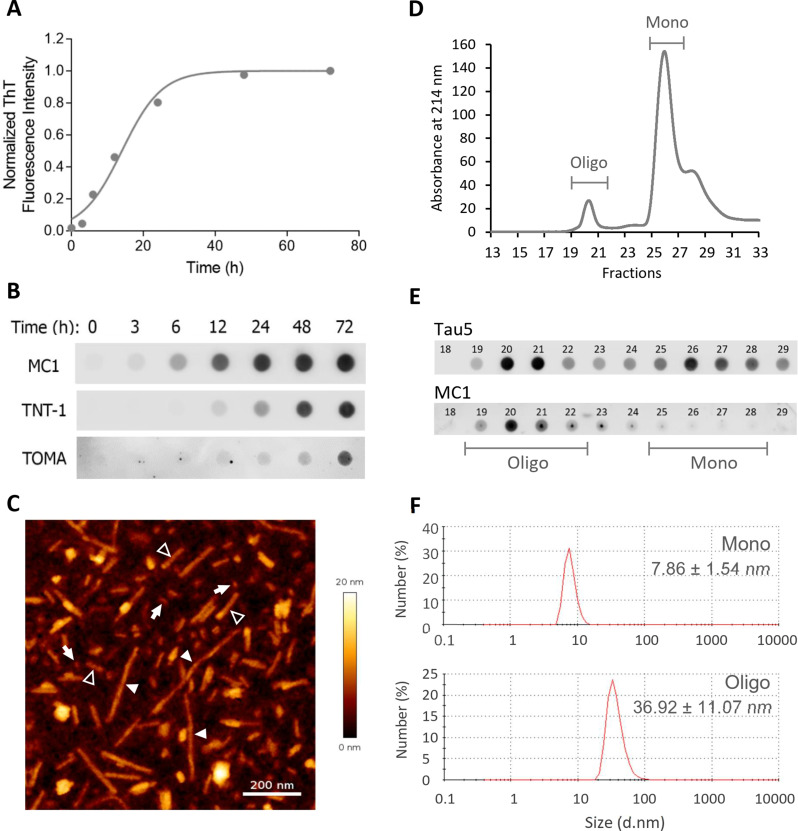
Characterization of recombinant 2N4R Tau aggregates. **A** Fibril formation kinetics of recombinant human 2N4R Tau by Thioflavin T (ThT) fluorescence, fitted with the Finke-Watzky model of two-step nucleation-autocatalysis. **B** Dot-blot analysis of Tau aggregates at different time points during the fibrillization process in A by three different conformation-sensitive Tau antibodies (MC1, TNT-1 and TOMA). **C** Atomic-force microscopic image of a mixture of aggregates after 72 h of fibrillization, including large fibrils (filled arrowheads), small fibrils (open arrowheads), and oligomers (arrows). Scale bar: 200 nm. **D** Size-exclusion chromatography (SEC) analysis of the soluble fraction of aggregates after removing insoluble fibrils by ultracentrifugation, showing the absorbance at 214 nm in the eluting fractions, including Tau oligomers (Oligo) and monomers (Mono). **E** Dot-blot analysis of SEC fractions in **D** using the antibodies Tau5 (total Tau) and MC1 (conformationally altered Tau). **F** Dynamic light scattering measurements of Mono and Oligo Tau showing the hydrodynamic size distribution of soluble Tau species obtained from SEC (d.nm: diameter in nanometers)

To study the uptake and intracellular accumulation of extracellular Tau (eTau), we generated amyloid aggregates from recombinant 2N4R Tau via heparin-induced fibrillization in vitro. The formation of aggregates was confirmed using thioflavin-T (ThT) as an indicator of β-sheet conformation (Fig. 1A). The pathological relevance of these aggregates was confirmed by dot-blot immunoassay using conformational Tau antibodies, including MC1 (pathological Tau conformation), TNT-1 (pathological misfolded Tau), and TOMA (oligomeric Tau). The heparin-induced Tau aggregates were recognized by all conformational Tau antibodies at different stages of the fibril formation process (Fig. 1B). This figure lacks comparative value due to the different sensitivity of antibodies toward their epitopes.

Atomic force microscopy (AFM) of Tau after 72 h of fibrillization revealed the formation of different aggregated structures, including long and short fibrils as well as oligomers (Fig. 1C). The soluble aggregates were analyzed via size exclusion chromatography (SEC) followed by immunoassay after the removal of insoluble fibrils with sedimentation. The results showed a peak of high molecular weight species reactive for both total Tau antibody (Tau5) and MC1, indicating the presence of soluble oligomeric species (Fig. 1D and E). Dynamic light scattering measurements of SEC fractions showed that the average hydrodynamic size of monomers and oligomers were 7.86 ± 1.54 nm and 36.92 ± 11.07 nm, respectively (Fig. 1F). This is in accordance with previous studies showing an average size distribution of ~ 8–12 nm for Tau Monomers and ~ 40–55 nm for oligomers [12, 39, 40].

### Extracellular tau aggregates accumulate more rapidly inside neurons than native Tau Monomers

**Fig. 2:**
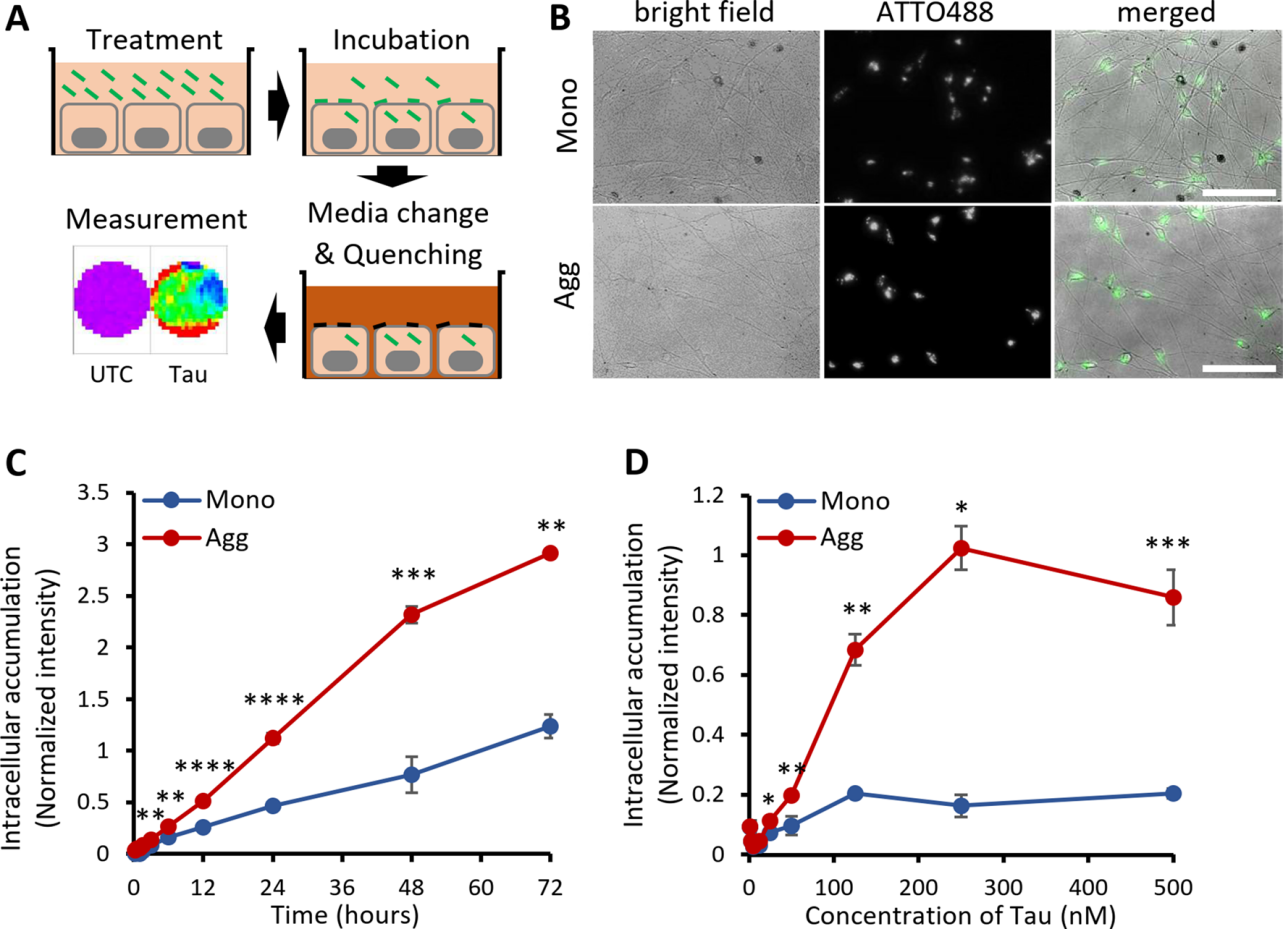
Extracellular Tau aggregates accumulate more than monomers in human iPSC-derived neurons. **A** Schematic representation of the uptake and accumulation assay. First, cells were treated with fluorescently ATTO488-labeled Tau. After a defined incubation time, the culture medium was changed to a quenching medium to eliminate the extracellular but not the intracellular fluorescence. Finally, a fluorescence plate reader quantified the well surface fluorescence in a 96-well–plate with a transparent bottom. UTC: untreated control. **B** Live images of cells (DIV 15) treated with 100 nM ATTO488-labeled Tau Monomers (Mono) or aggregates (Agg) for 24 h in the presence of the quencher. Scale bar: 100 μm. **C** Time-dependent uptake of 100 nM ATTO488-labeled Tau Monomers and aggregates, quantified on a fluorescence plate-reader in the presence of the quencher. **D** Concentration-dependent uptake of ATTO488-labeled Tau Monomers and aggregates (monomer equivalent) after 24 h on a fluorescence plate-reader in the presence of the quencher. Fluorescence values are normalized by dividing by the background. Error bars represent SD; *n* = 3 per experimental condition. One-way ANOVA followed by post-hoc test; ns: not significant, *p < 0.05, **p < 0.01, ***p < 0.001, ****p < 0.0001 vs. Mono

To monitor Tau’s neuronal uptake and intracellular accumulation, we developed an assay using recombinant monomeric and aggregated Tau in iPSCNs. We have previously shown that small molecule-induced neural precursor cell lines converted from iPSCs have an elevated expression of neuronal markers from day 10 of differentiation via NGN2 overexpression [33, 34]. As illustrated in Fig. 2A, the cortically differentiated neurons in culture were incubated with the fluorescently labeled Tau (Tau-ATTO488 NHS ester). Before measurement, the differentiation media was changed to a low-fluorescence medium containing a cell impermeable fluorescence quencher, as a similar approach described before [41]. The cell-impermeable fluorescence quencher eliminates extracellular signals, including the fluorescence signal of Tau species that interacts with the extracellular side of the cell membrane. Imaging confirmed the presence of fluorescent Tau protein only in association with the cell body or neurites (Fig. 2B), validating proof of concept.

iPSCNs were treated extracellularly with native Tau Monomers or heparin-induced aggregates to compare their neuronal accumulation during 72 h of exposure. Measuring the intracellular fluorescence as an indicator of internalized protein quantity revealed a significantly higher rate of intracellular accumulation for aggregates compared to monomers (Fig. 2C). Likewise, incubating the iPSCNs for 24 h with increasing concentration of extracellular Tau (eTau) revealed distinct saturation levels, which was much higher for aggregates than monomers (Fig. 2D). To enable comparability, aggregates were made from the same pool of labeled monomers to obtain identical labeling efficiency, as described in the method section. To ensure that the higher signal from aggregates is not related to an increase of fluorophores’ brightness in conformationally changed aggregates, we compared the fluorescent intensity of monomers and aggregates after treatment with an unfolding reagent, Guanidine Hydrochloride (GuHCl). Our result showed that exposure to GuHCl completely disaggregated Tau based on ThT fluorescence and increased the fluorescence intensity of ATTO-488 in monomers and aggregates by ~ 10% and 90%, respectively (Suppl. Fig. S1, Additional file 1). The increase of ATTO fluorescence in Monomers might be due to a slight change in the probe environment in the presence of 0.5% GuHCl. A significant increase in ATTO fluorescence intensity in aggregates compared to monomers suggests that the compact structure of aggregates may lead to a quenching effect on the probes. Thus, the higher fluorescence intensity of aggregates inside the cells is not associated with the fluorophore properties since, with the same labeling efficiency, aggregates are even less bright than monomers.

In order to test whether the uptake and accumulation of Tau aggregates is a specific cellular process or an unspecific endocytic event for any β-sheet containing protein aggregates, bovine serum albumin (BSA) was fluorescently labeled (BSA-CF488A) and fibrillized into amyloid aggregates. The formation of aggregates was confirmed by the Proteostat aggregation assay kit (Suppl. Fig. S2A, Additional file 1). iPSCNs were treated with fluorescently labeled BSA aggregates and monomers for 24 h. After removing the treatment and washing the cells, widespread fluorescent puncta were observed before the addition of the quencher, indicating a high cellular interaction of BSA monomers and aggregates. However, in the presence of a quencher, almost all fluorescence signals were eliminated (Suppl. Fig. S2B, Additional file 1). The absence of intraneuronal accumulation of BSA amyloid aggregates suggests that internalizing Tau aggregates in neurons is not a passive or nonspecific process.

### Generation and characterization of various tau aggregated species

**Fig. 3:**
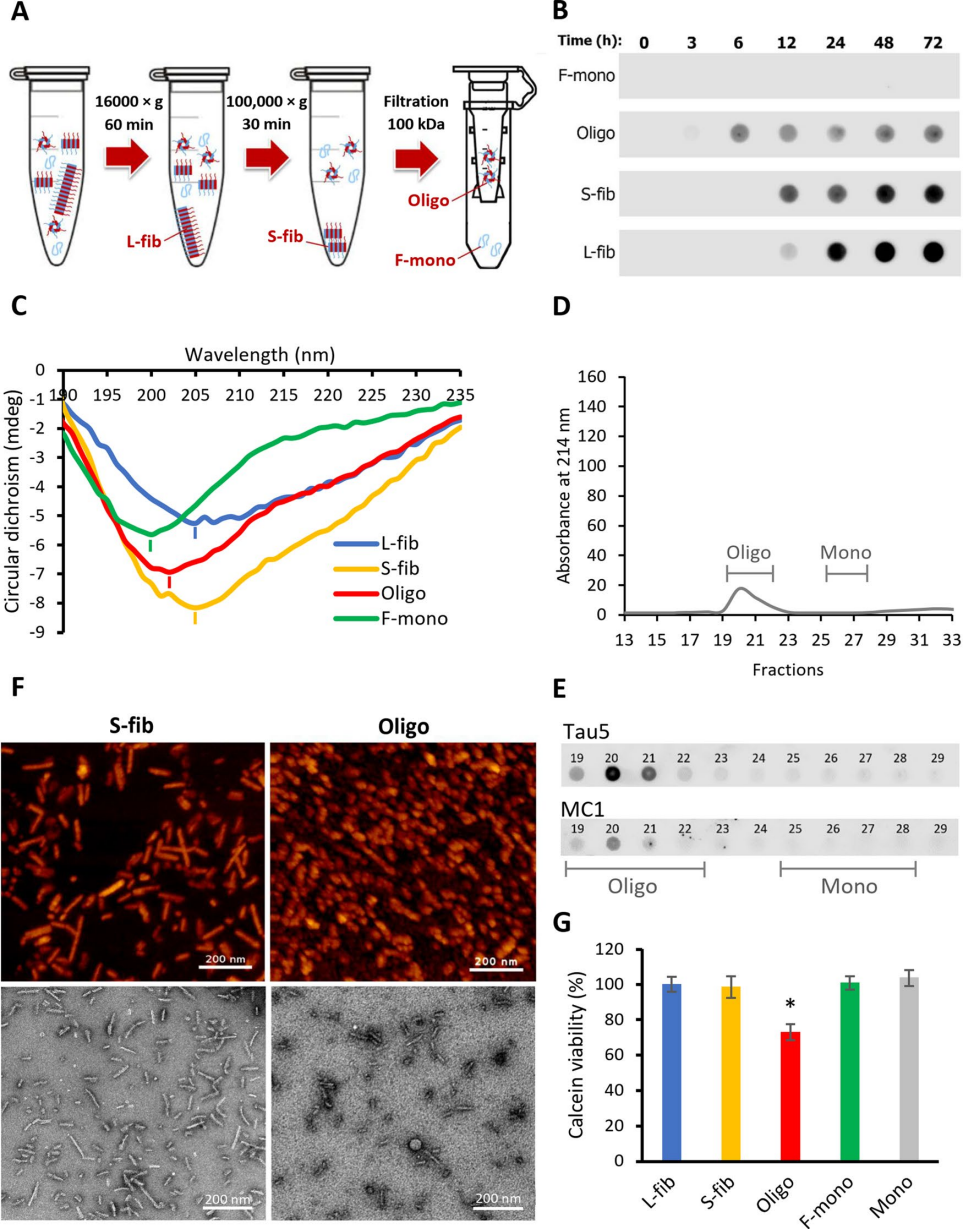
Characterization of different biochemical fractions of recombinant 2N4R Tau aggregates. **A** Schematic representation of the fractionation procedure including two sequential centrifugations of low (16,000 ×g) and high (100,000 ×g) gravitational force to sediment large insoluble fibrils (L-fib) and small fibrils (S-fib), respectively, followed by a 100 kDa filtration step separating the soluble oligomers (Oligo) that are retained on the filter from fibrillization-derived monomers (F-mono) that pass through the filter. **B** Dot-blot analysis of the content of fibrillization mixture and fractions during the fibrillization process using MC1 conformational antibody. **C** Circular dichroism spectrum showing the conformational status of Tau in each fraction (minimum of peaks showed with short lines). mdeg: millidegrees. **D** Size-exclusion chromatography (SEC) spectrum of Oligo fraction showing the absorbance at 214 nm of the eluting fractions with lines indicating the expected fractions for oligomers (Oligo) and monomers (Mono). **E** Dot-blot analysis of SEC fractions in D using the antibodies Tau5 (total Tau) and MC1 (conformationally altered Tau). **F** Atomic force microscopy (upper row) and transmission electron microscopy (lower row) images of small fibrils and soluble aggregates or oligomers. **G** Viability of iPSC-derived neurons treated for 24 h with 250 nM of either the Tau fractions mentioned above or recombinant Tau Monomers (Mono). Error bars represent SEM; *n* = 3 per experimental condition. One-way ANOVA followed by post-hoc test; *p < 0.05 vs. Mono

Various structures of Tau aggregated species have been identified in patient-derived samples [42]. Likewise, as depicted with AFM imaging (Fig. 1C), the heparin-induced Tau aggregated mixture was composed of different tau structures. To clarify more specifically the role of each species in the higher neuronal accumulation of Tau aggregates compared to monomers, we isolated and characterized four different Tau species from the aggregated mixture. As illustrated in Fig. 3A, using a stepwise procedure, Tau species were separated based on their biophysical properties, including molecular weight and size, by centrifugation, ultracentrifugation, and ultrafiltration.

The kinetics of various Tau aggregated species formation during the fibril formation process was studied by combining fractionation and dot-blot analysis (Fig. 3B). While the fibrillization-derived monomer fraction (F-mono) showed no reactivity toward MC1 conformational antibody, immunoreactivity appeared for the soluble oligomeric fraction (Oligo) from 6 h after the beginning of fibrillization, for the small fibrils fraction (S-fib) after 12 h, and for the large fibrils fraction (L-fib) mainly after 24 h. This data indicates that the fractionation successfully separated the species of aggregates that form gradually during the fibrillization process.

Circular dichroism (CD) was carried out to study conformational differences between the species. The peak minimum at ~ 200 nm for F-Mono confirmed a pure random coil structure, as expected for a natively unfolded protein [43], while a redshift to 205 nm for fibrils indicates the presence of β-sheet conformation (Fig. 3C). The lack of a clear β-sheet spectrum is due to the presence of various conformations, including β-sheet in the core and random coil in the N- and C-terminal flanking region of fibrils. A similar CD spectrum was reported by comparing 2N4R Tau Monomers and fibrils [12, 28]. Oligo fraction with a minimum at 203 nm was located between monomers and fibrils, suggesting an intermediate structure with less β-sheet content than fibrils.

A gradient centrifugation of iodixanol was performed to compare the density of species in soluble and insoluble fractions, which showed a separation between L-fib, S-fib, and soluble species that contain monomers, multimers, and oligomers (Suppl. Fig. S3, Additional file 1). Some overlaps between the species can be observed, which might be partially related to the limitations of this technique. For evaluating the purity of oligomeric fraction, the SEC analysis was performed, which showed absence of a monomeric peak (Fig. 3D). Dot-blot analysis of SEC fractions by Tau5 and MC1 confirmed the lack of monomers contamination in Oligo fraction (Fig. 3E). To decipher further the structural differences between fibrils and oligomers, AFM and transmission electron microscopy (TEM) were implemented, which revealed the morphological differences (Fig. 3F). In contrast to fully elongated fibrillar structures in S-fib, Oligo fractions were mainly composed of smaller aggregates that appear spherical in AFM and rod-shape to spherical in higher resolution of TEM. Finally, the viability assessment of cells treated with different fractions showed significant toxicity of Oligo fraction only at a high concentration of 250 nM and after 24 h of treatment, while the other fractions and native Tau Monomers showed no impact on cell viability (Fig. 3G).

### Small fibrils and oligomers efficiently accumulate inside neurons, enter cytosol and seed intracellular aggregation

In order to find Tau species involved in higher intracellular accumulation of aggregates versus monomers (Fig. 2C and D), aggregates of Tau were fluorescently labeled after the fibrillization and then fractionated into four different species, as mentioned before. CD analysis of labeled and unlabeled fractions showed a similar negative peak, suggesting that the labeling did not affect the conformational features of the Tau species (Suppl. Fig. S4, Additional file 1). The degree of labeling was calculated to estimate the comparability of each fraction based on the fluorescence intensity (Suppl. Table S1, Additional file 1). Approximately one label per 2.14–2.39 monomeric Tau in the fractions indicated a close range of diversity and ensured the comparability for later analysis.

**Fig. 4:**
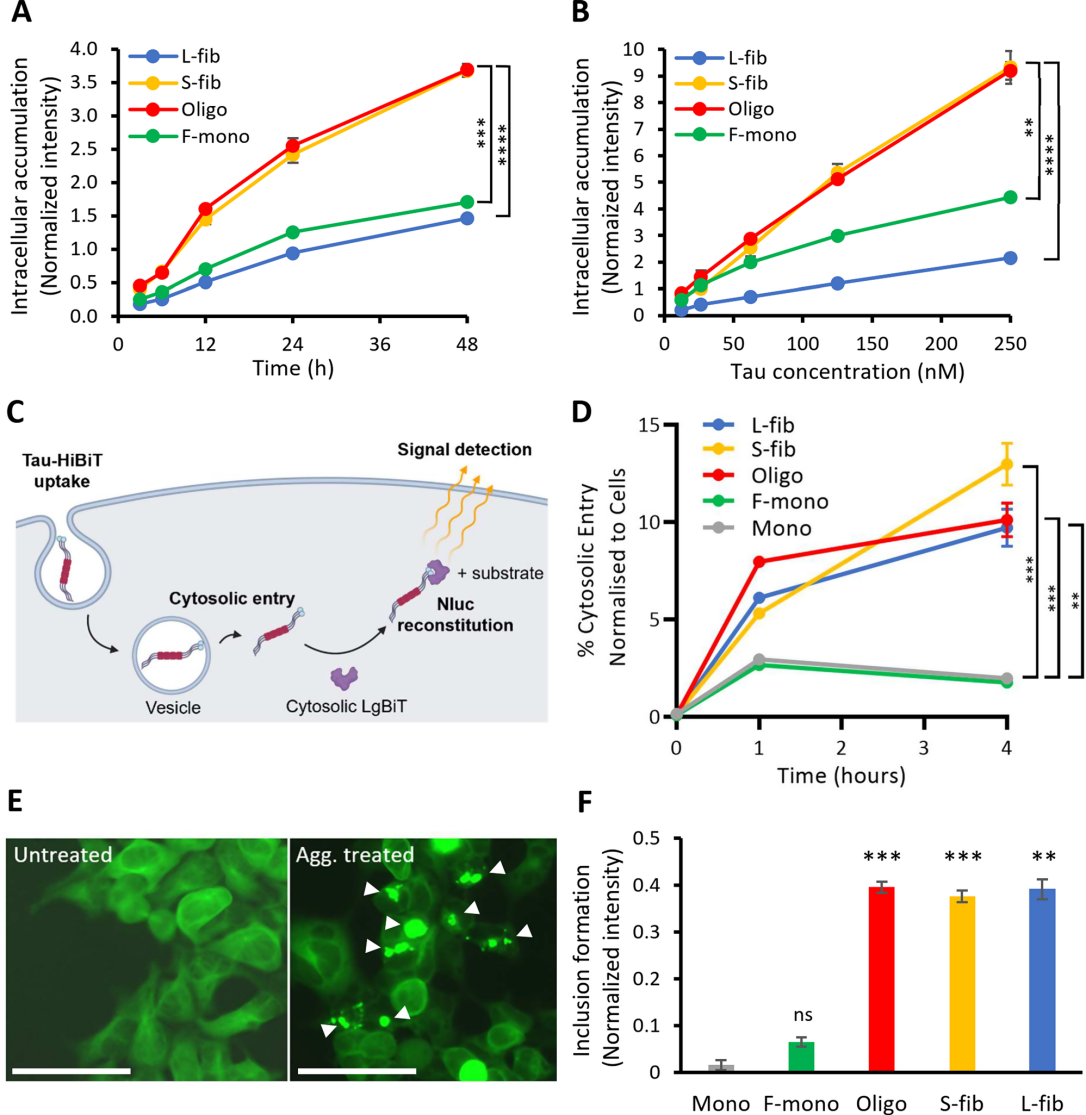
Intracellular accumulation, escape to the cytosol and endogenous aggregation of various recombinant 2N4R Tau species. **A** The kinetics of intracellular accumulation of different Tau species, including large fibrils (L-fib), small fibrils (S-fib), oligomers (Oligo), fibrillization-derived monomers (F-mono), and recombinant monomers (Mono) within 48 h with 100 nM labeled Tau species. **B** Titration curve for intracellular accumulation of different Tau species after 20 h of incubation. Error bars represent SD; *n* = 3 per experimental condition. One-way ANOVA followed by a post-hoc test; ns: not significant, **p < 0.01, ***p < 0.001, ****p < 0.0001. **C** Schematic of the Tau entry assay. 0N4R P301S-Tau-HiBiT assemblies were added to cells expressing cytosolic LgBiT. Uptake of Tau assemblies may lead to cytosolic entry, resulting in Tau-HiBiT-mediated Nluc reconstitution by LgBiT binding. The addition of cell-permeable substrate results in the Nluc-mediated production of photons, which are readily quantifiable. Cytosolic entry is, therefore, proportional to the detected luminescent signal. **D** Percent of Tau-HiBiT that enters the cytosol of GPLN neurons following exposure to 2 ug/ml Tau-HiBiT monomers or Tau aggregated species (L-fib, S-fib, Oligo, Mono). Error bars denote SD. *n* = 3 per experimental condition. **p < 0.01; ***p < 0.001 by two-way ANOVA with Dunnett’s multiple comparisons. **E** Fluorescence microscopic images of HEK293-biosensor cells expressing P301S Tau-venus, either left untreated or treated with 200 nM unlabeled Tau small fibrils (S-fib). Arrows showing the inclusions of endogenous P301S Tau-venus. Scale bar: 61.7 nm. **F** Fluorescence analysis of cells treated with 200 nM Tau fractions and monomer using a fluorescence plate reader. Error bars represent SEM; *n* = 3 per experimental condition. One-way ANOVA followed by a post-hoc test; ns: not significant, **p < 0.01, ***p < 0.001 vs. Mono

The kinetics and titration analysis revealed a significantly higher eTau S-fib and Oligo accumulation rate than L-fib and F-mono in iPSCNs (Fig. 4A and B). This suggests efficient neuronal internalization of small and intermediate aggregates compared to Tau Monomers or large aggregates. The uptake and accumulation of eTau species were also studied in Lund human mesencephalic (LUHMES) neurons as a different model of human neurons [35]. Kinetics and titration analysis confirmed the previous finding in LUHMES neurons (Suppl. Fig. S5A and S5B, Additional file 1). However, the rate of cell accumulation for F-mono was markedly lower compared to L-fib. Comparisons of intracellular accumulation of Tau in the two neuronal models showed that native monomers are accumulating at a much higher rate in iPSCNs than LUHMES neurons (Suppl. Fig. S5C, Additional file 1) while aggregates uptake (S-fib) in both cell models had a similar rate (Suppl. Fig. S5D Additional file 1).

Based on the prion-like spreading hypothesis, potent propagating seeds are required to internalize neurons, enter the cytosol, and induce endogenous aggregation [44]. Thus, before proceeding to further mechanistic analysis of the uptake and accumulation process, the capacity of fractions as a prion-like seed was evaluated. A live-cell neuronal entry assay was carried out, as described before [37], by using a split luciferase called NanoLuc (Nluc) composed of a large 18 kDa subunit (LgBiT) and a small 11 amino acid peptide (HiBit) forms a complementation reporter [38]. HiBiT-tagged Tau was added to the extracellular medium of primary mouse neurons expressing LgBiT. Tuck et al. previously showed that human and mouse neurons had similar Tau uptake dependencies [37]. In this model, the intracellular luminescence only appears in the presence of substrate when the extracellular Tau-HiBiT meets the endogenous LgBiT in the cytosol and reconstitutes the complete Nluc (Fig. 4C). Here, we fibrillized Tau-HiBiT and fractionated as described before, then LgBiT expressing primary neurons were treated with fractions for 4 h, before the measurement. As shown in Fig. 4D, all aggregated fractions were similarly capable of entering the cytosol and accumulating more than monomeric species inside neurons.

To examine the seeding potential of the Tau fraction, a HEK293 biosensor cell line with stable overexpression of 0N4R Tau containing P301S mutation that was C-terminally tagged with Venus fluorescence protein was implemented as described before [36]. Under physiological conditions, a microtubule-associated distribution of Tau was exhibited by the biosensor cells, while adding Tau aggregates induced the formation of fluorescent puncta, which are easily traceable by total fluorescence measurement due to a brighter signal (Fig. 4E, and lower magnifications pictures in Suppl. Fig. S6, Additional file 1). The fluorescence measurement with a plate reader revealed a significantly higher fluorescence intensity for cells treated with L-fib, S-fib, and Oligo than those treated with F-mono and Mono, confirming their capacity for seeding endogenous native Tau (Fig. 4F).

### Competition assay indicates a distinct mechanism for intraneuronal accumulation of monomers and aggregates

**Fig. 5:**
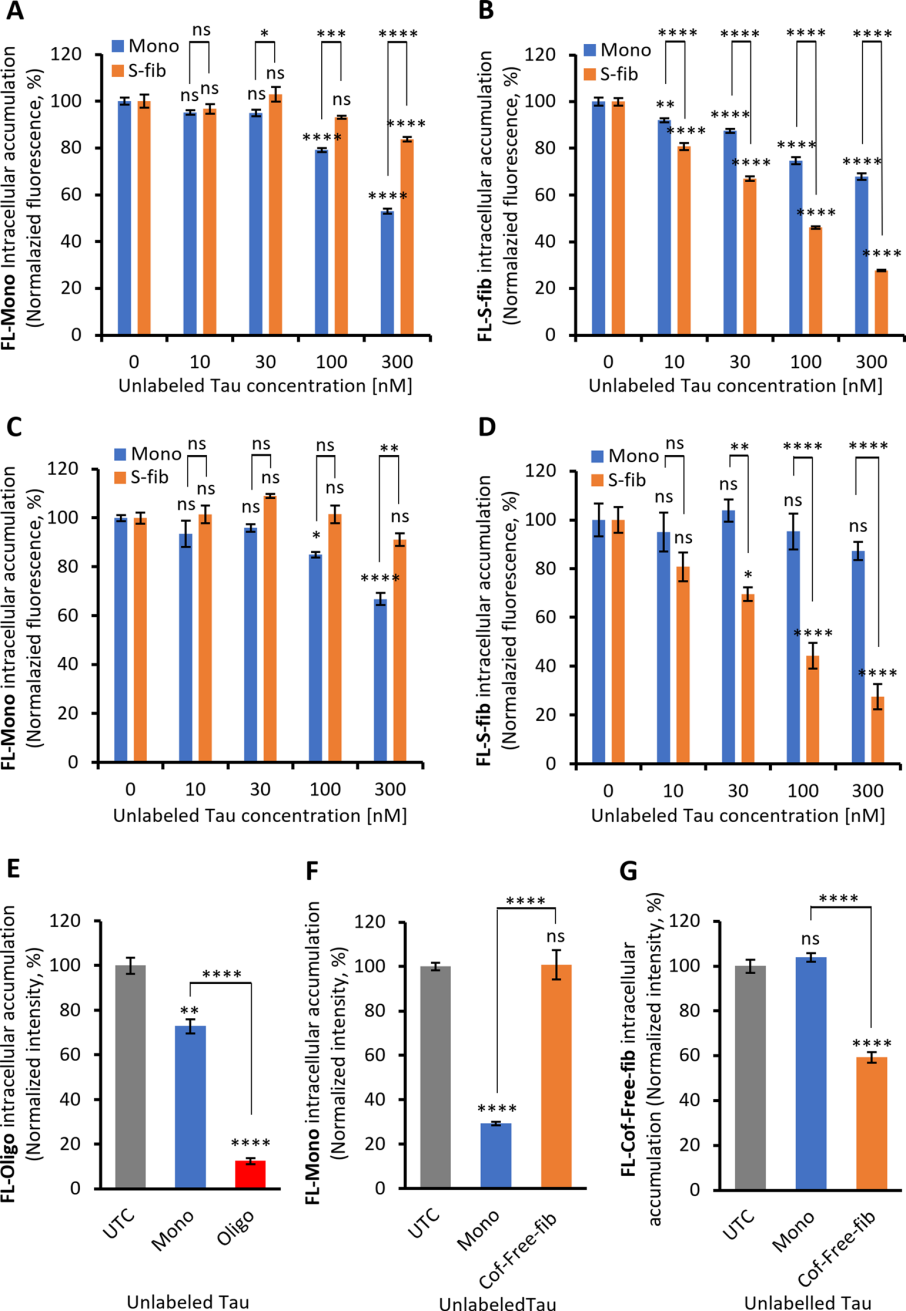
The competition between labeled and unlabeled extracellular Tau Monomers and fibrils for intraneuronal accumulation. The uptake and accumulation of 50 nM fluorescently labeled monomers (FL-Mono, A) and small fibrils (FL-S-fib, B) in the presence of increasing concentrations of unlabeled Tau Monomers (Mono, blue bars) and small fibrils (S-fib, orange bars) after 20 h of incubation in iPSCNs (**A**,** B**), and in LUHMES neurons (**C**,** D**). Significance was calculated by comparing Mono and S-fib at each concentration versus “0” and versus each other. **E** The neuronal accumulation of fluorescently labeled oligomers (50 nM) in iPSCNs neurons after 20 h of incubation in the presence of a 5-fold higher concentration of unlabeled monomers and oligomers (250 nM). **F** The uptake and accumulation of 25 nM FL-Mono in the presence of a 4-fold higher concentration of unlabeled Tau Mono and cofactor-free fibrils (Cof-free-fib) after 20 h of incubation. **G** The uptake and accumulation of 25 nM fluorescently labeled Cof-free-fib (FL-Cof-free-fib) in the presence of a 4-fold higher concentration of unlabeled Tau Mono and Cof-free-fib after 20 h of incubation. Significance compared to the untreated control (UTC). Error bars represent SEM; *n* ≥ 3 independent experiments per experimental condition. Two-way ANOVA followed by Tukey post-hoc test; ns: not significant, *p < 0.05, **p < 0.01, ***p < 0.001, ****p < 0.0001

Due to the different rates of neuronal accumulation between monomers and aggregates, we hypothesized that the accumulation pathways of these species are distinct. Thus, we tested this by studying the competition between native monomeric and pure Tau fractions for intraneuronal accumulation. Since the uptake and accumulation of L-fibs was less than that of other species, and since the large, insoluble structure of L-fib reduces the chance of being the spreading species, we mainly focused on S-fib and Oligo for the subsequent studies. iPSCNs were treated with a constant concentration (50 nM) of fluorescently labeled Tau Mono (FL-Mono) or S-fib (FL-S-fib) and concomitantly treated with an increasing concentration of unlabeled Tau Mono or S-fib. Measuring the intracellular fluorescence intensity after about 20 h revealed that both unlabeled Mono and S-fib significantly reduced the accumulation of FL-Mono (Fig. 5A); however, the impact of unlabeled Mono was significantly more than the impact of unlabeled S-fib at 10 nM and higher concentrations. This suggests the competition between monomeric species is higher than the competition between monomers and fibrils. On the other hand, the accumulation of FL-S-fib in iPSCNs significantly declined in the presence of both unlabeled Mono and S-fib (Fig. 5B); however, the impact of S-fib was significantly more than Mono at all concentrations. This confirms a smaller cross-species competition for Tau accumulation than the competition between similar species, suggesting a distinct but overlapping mechanism of Tau accumulation between monomers and fibrils in iPSCNs.

Competition assay was also performed in LUHMES neurons, where we surprisingly found no cross-species competition. Treatment with the highest concentration of unlabeled S-fib was ineffective on the accumulation of FL-Mono (Fig. 5C). Similarly, the highest concentration of unlabeled Mono was ineffective on the uptake of FL-S-fib (Fig. 5D). This suggests a distinct accumulation mechanism for eTau Mono and S-fib in LUHMES neurons.

Similar to S-fib, the accumulation of FL-Oligo in iPSCNs was significantly reduced in the presence of a five-fold excess of either unlabeled Mono and Oligo (Fig. 5E). The effect of Oligo was significantly higher than Mono, suggesting a greater competition between similar eTau species rather than different species.

As heparin is a known inhibitor of Tau uptake [16] and since heparin was used as an inducer of recombinant Tau fibrillization, we tested Tau aggregates, which were produced without any co-factor like heparin to eliminate the potential role of heparin in our uptake competition assay [28]. Our result showed no cross-species competition for intraneuronal accumulation between Mono and co-factor-free fibrils (Cof-free-fib) in iPSCNs (Fig. 5F and G). This data confirms that the uptake competition between Tau fibrils is not associated with the inhibitory impact of heparin.

Altogether, a partial competition for intraneuronal accumulation between the eTau Monomers and heparin-induced aggregates in iPSCNs and a lack of competition in LUHMES neurons and with Cof-free-fib in iPSCNs suggest a distinct mechanism of uptake and accumulation between physiological and pathology-relevant Tau species.

### Inhibition of endocytic pathways similarly alters neuronal accumulation of extracellular Tau Monomers and small fibrils in iPSCNs

**Fig. 6:**
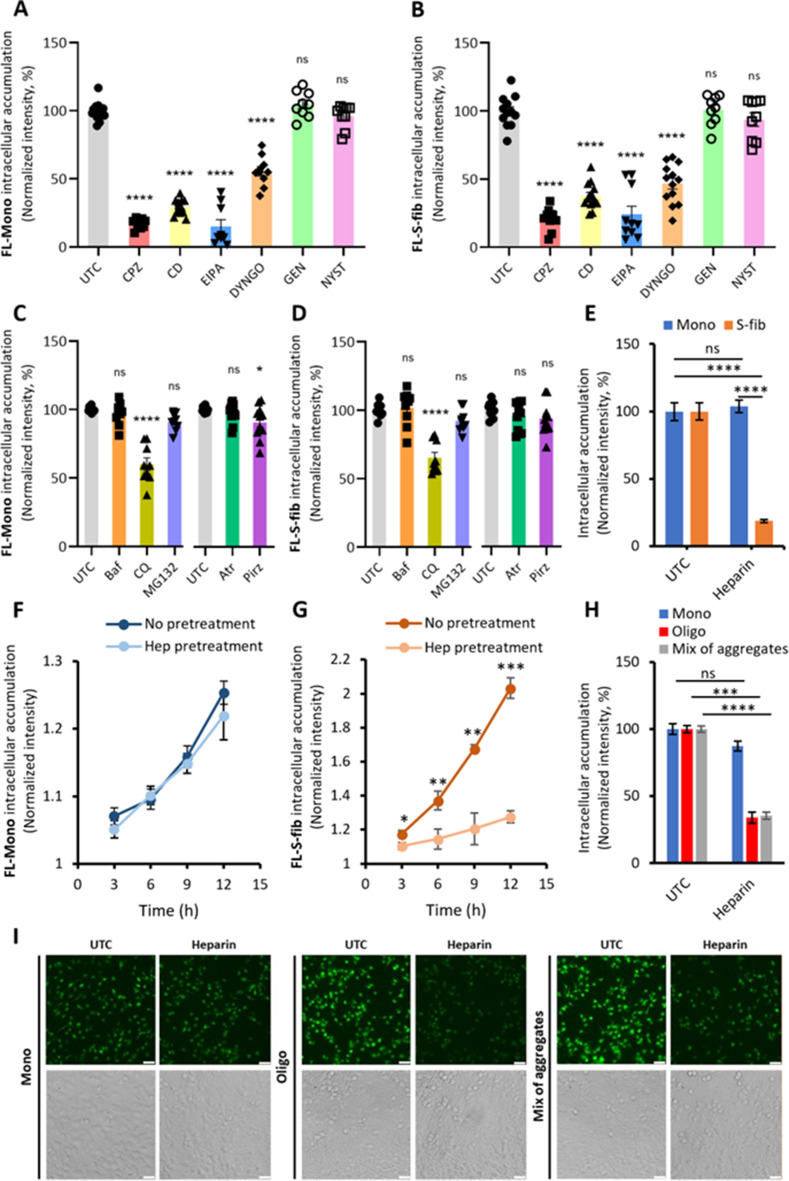
The impact of small molecule inhibitors on the intracellular accumulation of Tau. The intracellular level of Tau in cells left untreated as control (UTC) or treated with 50 µM Chlorpromazine (CPZ), 20 µM Cytochalasin D (CD), 30 µM 5-N-ethyl-N-isopropyl amiloride (EIPA), 75 µM Dyngo-4a (DYNGO), 200 µM Genistein (GEN), or 10 µM Nystatin (NYST) for 30 min before incubation with **A** fluorescently labeled monomers (FL-Mono), and **B** fluorescently labeled small fibrils (FL-S-fib), both at 250nM concentration for 3 h (exceptionally, EIPA were present during the incubation with Tau). Error bars represent SEM; *n* = 9–14. One-way ANOVA ****p < 0.0001. Fluorescence measurement of cells treated with 25 nM fluorescently labeled Tau **C** FL-Mono, and **D** FL-S-fib in the presence of 100 nM bafilomycin A1 (Baf), 30 µM chloroquine (CQ), 100 nM MG132, 200 µM Atropine (Atr) or 20 µM Pirenzepine (Pirz) for 20 h. Error bars represent SEM; *n* = 9–14 independent experiments per experimental condition. One-way ANOVA ****p < 0.0001, *p < 0.05 vs. UTC. **E** Fluorescence analysis of iPSCNs treated with 25 nM labeled Tau Monomers and small fibrils for 20 h in the presence of 2 µM Heparin. Error bars represent SEM; *n* = 3. One-way ANOVA followed by post-hoc test; ****p < 0.0001 vs. UTC. ns: not significant. Kinetics of intracellular Tau accumulation in LUHMES neurons pre-treated with 100 µM Heparin for 2 h before exposure to **F** 250 nM fluorescently labeled Mono (FL-Mono) and **G** 150 nM fluorescently labeled-small fibrils (FL-S-fib). The significance was calculated between “No pretreat” and “Pretreat” at each time point (Only significant points were shown). Error bars represent SD. *n* = 3 per experimental condition. One-way ANOVA followed by posthoc test; *p < 0.05, **p < 0.01, ***p < 0.001 vs. “No pretreatment”. **H** Intracellular accumulation in LUHMES neurons pretreated with 100 µM Heparin for 2 h before 9 h treatment with 100 nM fluorescently labeled Tau Monomers (Mono), 50 nM Oligomers (Oligo), or 50 nM mixture of aggregates including large fibrils, small fibrils, and oligomers. Error bars represent SEM. *n* = 3 per experimental condition. One-way ANOVA followed by posthoc test; ***p < 0.001, ****p < 0.0001 vs. UTC. ns: not significant. **I** Representative images of H. Scale bar: 25 nm

To further investigate the cellular mechanism for eTau Monomers and aggregates’ distinct uptake and accumulation routes, iPSCNs were treated with small molecule inhibitors of different endocytic pathways (Table 2). Following a 30-minute exposure, inhibitors were washed away and neurons were treated with fluorescently labeled Tau Mono or S-fib for 3 h. The data revealed that Chlorpromazine (CPZ), Cytochalasin-D (CD), 5-(N-ethyl-N-isopropyl)-amiloride (EIPA), and Dyngo-4a (DYNGO) significantly reduced the uptake of both Mono and S-fib, but Genistein (GEN) and Nystatin (NYST) were ineffective (Fig. 6A and B). Representative images were shown in Suppl. Fig. S7A, Additional file 1. In parallel, we used a viability assay representing the cell density to monitor the effect of treatments on the cell population. We showed that, similar to Hoechst staining, the Calcein-AM viability assay is representative of cell density (Suppl. Fig. S8A to S8C, Additional file 1). None of the treatments had an impact on the cell viability (Suppl. Fig. S8D, Additional file 1). These results suggest clathrin-mediated endocytosis and micropinocytosis are involved in the uptake of Tau, and actin polymerization and dynamin function are necessary for this process.

To investigate the effect of other possible cellular mechanisms, iPSCNs were treated with small molecule inhibitors of protein degradation pathways and muscarinic receptors reportedly involved in the Tau uptake (Table 3). Measuring intracellular fluorescence after 20 h of co-treatment of FL-Tau and inhibitors revealed that only Chloroquine (CQ) significantly reduced the Tau uptake for both Mono and S-fib, while Bafilomycin and MG132 were ineffective (Fig. 6C and D). Among muscarinic receptor antagonists, only Pirenzepine mildly but significantly reduced the accumulation of Tau Mono in iPSCNs, but it was ineffective on the accumulation of Tau S-fib. Representative images were shown in Suppl. Fig. S7B, Additional file 1. None of the treatments were toxic to the cells (Suppl. Fig. S8E, Additional file 1). This suggests that protein degradation is not a critical factor in determining the amount of Tau in cells in a short time frame of < 20 h. The unexpected impact of CQ might be related to the interference with receptor recovery to the cell membrane, as the endolysosomal system is known to be involved in receptor recycling [45]. Despite a similar function of Baf A1 and CQ, the difference in their effect on Tau accumulation might be related to the different molecular mechanisms, as shown before [46, 47]. The mild differential impact of muscarinic receptors can not be a major mechanism in the noticeable differential accumulation of monomeric and aggregated Tau species.

Next, we tested the inhibition of HSPGs as the harbors for Tau-cell interaction during the internalization process [16]. iPSCNs were co-treated with FL-Tau Mono or S-fib along with heparin as a blocker of HSPGs for 20 h. As depicted in Fig. 6E, heparin significantly reduced the accumulation of S-fib Tau, while it was ineffective on Mono Tau. Representative images were shown in Suppl. Fig. S7B, Additional file 1. No change in viability was observed upon treatment with Heparin (Suppl. Fig. S8F, Additional file 1). This data was also reproduced and confirmed in LUHMES neurons (data not shown).

To further validate the role of HSPGs in the differential accumulation of extracellular monomeric and aggregated Tau species, we studied the impact of heparin pretreatment on the uptake of Tau to avoid the co-existence of both Tau and heparin in the media and to omit any unwanted inhibitory interaction in the soluble phase. Thus, after a 2-hour of heparin exposure, heparin-containing media was discarded and LUHMES neurons were washed to remove the residual free heparin in the extracellular medium before treating the cells with FL-Tau species. Measuring intracellular fluorescence during 12 h revealed no significant differences between untreated and heparin-pretreated cells in the accumulation of FL-Mono (Fig. 6F). However, the neuronal accumulation of FL-S-fib was significantly reduced in heparin-pretreated cells compared to non-pretreated cells (Fig. 6G). Moreover, we tested the effect of Heparin pretreatment on the uptake and accumulation of Tau Oligo and a mixture of aggregates, including L-fib, S-fib and Oligo (Fig. 6H and I). The results confirmed the inhibiting effect of heparin on the intracellular accumulation of all extracellular aggregated species. This data indicates that HSPGs are involved in the differential uptake and accumulation of eTau Monomers versus aggregates in human neurons.

We also used the pretreatment assay to study the competition dynamics between the same eTau species to omit the possibility of Tau-Tau interaction in the extracellular media. Thus, following a 2-hour exposure to Tau Mono or S-fib, Tau-containing media was discarded and LUHMES neurons were washed. Then, cells were treated with FL-Mono or FL-S-fib and the intracellular fluorescence was monitored at different time points during 24 h of incubation. Our result revealed that Mono pretreatment did not significantly impact the intracellular accumulation of FL-Mono (**Suppl. Fig. S9A**,** Additional file 1**). In contrast, 2 h of pretreatment with S-fib causes a significantly slower rate of accumulation of FL-S-fib in the early hours until 9 h after treatment (Suppl. Fig. S9B, Additional file 1). Interestingly, after 12 h of incubation with FL-S-fib, the rate of increase in fluorescence signal was similar between pretreated and non-pretreated cells. This suggests a recovery point where the cells can retrieve their capacity to internalize and accumulate eTau aggregates. This data further confirms mechanistic divergence in the intracellular accumulation between eTau aggregates and monomers.

**Table 2:** Description and mode of action for inhibitors of endocytic pathways

No.	Name	Function
1	Chlorpromazine (CPZ)	An inhibitor of clathrin-mediated endocytosis via binding to adaptor protein − 2 (AP-2) subunit [48]
2	Cytochalasin-D (CD)	An inhibitor of actin polymerization (49), which is involved in various endocytic processes [50]
3	5-(N-ethyl-N-isopropyl)-amiloride (EIPA)	An inhibitor of macropinocytosis, which interferes with Na^+^/H^+^ exchange [51]
4	Dyngo-4a (DYNGO)	An inhibitor of both dynamin I and II [52]
5	Genistein (GEN)	An inhibitor of tyrosine kinases involved in caveolar-mediated endocytosis [53]
6	Nystatin (NYST)	A Cholesterol-chelating that disrupts lipid rafts and inhibits clathrin-independent, caveolar-mediated endocytosis [54]
7	Bafilomycin A1 (Baf)	An inhibitor of vacuolar ATPase that prevents lysosomal acidification, inhibiting the activation of lysosomal proteases [55]
8	Chloroquine (CQ)	An inhibitor of autophagic flux by blocking the fusion of autophagosomes with lysosomes [56]

**Table 3:** Description and mode of action for inhibitors of protein degradation pathways and muscarinic receptors

No.	Name	Function
1	Bafilomycin A1 (Baf)	An inhibitor of vacuolar ATPase that prevents lysosomal acidification, inhibiting the activation of lysosomal proteases (55)
2	Chloroquine (CQ)	An inhibitor of autophagic flux by blocking the fusion of autophagosomes with lysosomes (56)
3	MG132	An inhibitor of proteasome activity by blocking the proteolytic activity of the 26 S proteasome complex (57)
4	Atropine (Atr)	A non-selective antagonist of the muscarinic receptor that inhibits Tau uptake (58)
5	Pirenzepine (Pirz)	An antagonist of the M1 muscarinic receptor that inhibits Tau uptake (58)

### Knockdown of molecular mediators of tau uptake differentially modulates the intracellular accumulation of Tau Monomers and aggregates

**Fig. 7:**
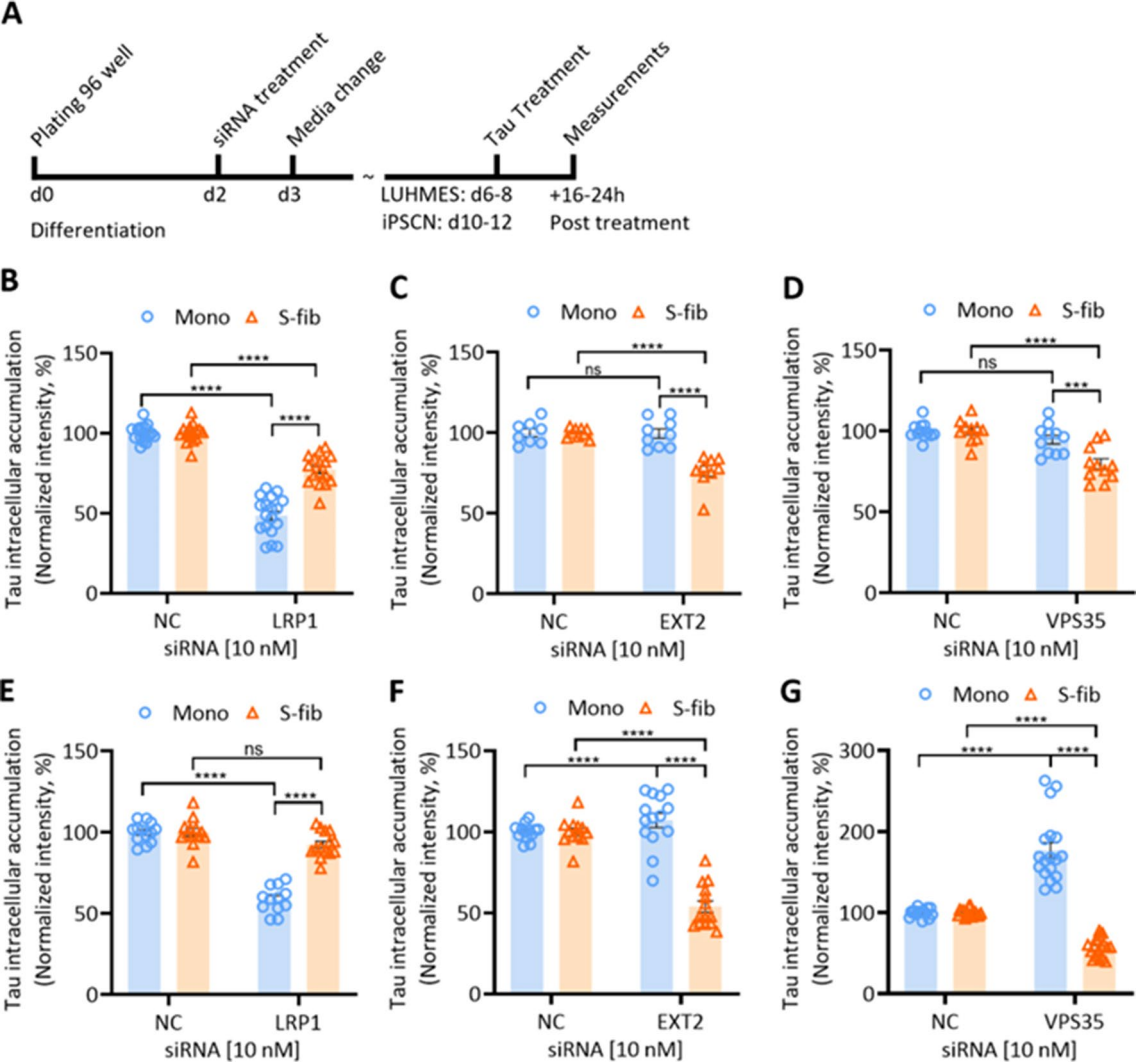
siRNA-mediated downregulation of some molecular mediators differentially impacts the intracellular accumulation of Tau Monomers and small fibrils. **A** Timeline of the experimental scheme. iPSC-derived neuronal progenitor cells or LUHMES cells were seeded in 96 well-plates in the differentiation medium. Cells were treated with 10nM siRNA at day 2 of differentiation. iPSCNSs and LUHMES neurons were treated with fluorescently labeled Tau at days 10 to 12 and 6 to 8, respectively. Fluorescence measurements were implemented after 16 to 24 h of treatment. Intracellular accumulation of labeled Tau Monomers and small fibrils were shown in **B**,** C**,**D** iPSCNs, and **E**,** F**,**G** LUHMES neurons that were treated with siRNAs of LRP1, EXT2, and VPS35. Error bars represent SEM; *n* = 9–18. One-way ANOVA ***p < 0.001, ****p < 0.0001. Error bars represent SEM. NC: negative control siRNA

In the final step to verify the differential uptake and accumulation of eTau Monomers and aggregates at the molecular level in our cell models, we used an in vitro knockdown approach for a selected list of genes relevant to Tau uptake and cell sorting pathways. As illustrated in Fig. 7A, iPSCNs and LUHMES neurons were treated with siRNA on day two of differentiation. Tau uptake has been analyzed after 16–24 h of treatment with labeled monomers and aggregates. For both iPSCNs and LUHMES neurons, we found three genes with differential regulation of Tau Mono and S-fib, including low-density lipoprotein receptor-related protein 1 (LRP1), exostosin glycosyltransferase 2 (EXT2) and vacuolar protein sorting-associated protein 35 (VPS35) (Fig. 7B − 7G). Knockdown characterization by western blot and representative images were shown in Suppl. Fig. S10A – S10D, Additional file 1. There was no change in cell viability upon these genes’ down regulations (Suppl. Fig. S10E and S10F, Additional file 1).

The surface receptor LRP1 was previously described as the master regulator of Tau uptake [19]. In this study, the knockout of LRP1 in iPSCNs resulted in almost complete inhibition of Tau Monomers, although a partial inhibition of fibril uptake was reported [19]. Likewise, our data showed that the knockdown of LRP1 in iPSCNs significantly reduced the intracellular accumulation of both Mono and S-fib. However, the reduction of S-fib uptake was significantly less than Mono (Fig. 7B). In LUHMES neurons, LRP1 knockdown reduced the uptake and accumulation of Mono, but surprisingly, it was ineffective in S-fib uptake (Fig. 7E). This data suggests that in LUHMES neurons, the uptake of eTau S-fib, unlike Mono, is independent of LRP1.

EXT2 is a member of the exostosin glycosyltransferase family, which is involved in the synthesis of HSPGs, and its knockdown has been reported to reduce the uptake of Tau [17, 18]. As we showed with heparin, HSPG inhibition only reduced the uptake and accumulation of eTau aggregates but not monomers. Similarly, EXT2 downregulation significantly reduced the intracellular accumulation of S-fib but not for Mono in both iPSCNs and LUHMES neurons (Fig. 7C and F). This result further confirms the role of HSPGs in the uptake of eTau aggregates but not monomers in human neurons.

In our small hypothesis-based screen, we also found differential regulation of uptake and accumulation of eTau Mono and S-fib in neurons with VPS35 downregulation. In iPSCNs, the knockdown of VPS35 significantly reduced the accumulation of S-fib while not affecting Mono (Fig. 7D). In LUHMES neurons, VPS35 downregulation led to a significant reduction of S-fib but interestingly intensively increased the accumulation of Mono (Fig. 7G). As VPS35 is a critical component of the retromer complex, our data suggest that this complex might be a critical regulator of Tau uptake and intracellular accumulation. Further analysis would be necessary to determine the exact role of the retromer complex in the eTau transfer and its cellular distribution.

To examine the role of LRP1 and HSPG downregulation on the uptake of other types of Tau aggregates, we repeated the experiment with Oligo and Cof-free fib in LUHMES neurons. Similar to S-fib, neuronal accumulation of Oligo was not sensitive to LRP1 downregulation, but EXT1 knockdown significantly reduced Oligo’s accumulation by about 50% (Suppl. Fig. S11A, Additional file 1). The intraneuronal accumulation of Cof-free fib in LUHMES neurons was similar to the Oligo fraction of heparin-induced Tau aggregates. No toxicity was observed in the abovementioned conditions (Suppl. Fig. S11B, Additional file 1). Moreover, to make sure that the effect is not exclusive to isolated fractions, we tested a mixture of fractions, including L-fib, S-fib, and Oligo with the same siRNAs, and the result confirmed previous observations (Suppl. Fig. S11C – S11E, Additional file 1). This data further confirmed the role of HSPGs but not LRP1 in the uptake and accumulation of eTau aggregates in LUHMES neurons.

As we showed in Suppl. Fig. S5C and S5D, the accumulation of Tau S-fib is similar between iPSCNs and LUHMES neurons, while the Mono accumulation is intensively different. Based on the result of the siRNA study, we hypothesized that this difference could be due to the different expressions of LRP1. Our western blot analysis revealed that the expression of LRP1 in iPSCNs is about twenty times higher than LUHEMS neurons (Suppl. Fig. S12A – S12B, Additional file 1), which explains the different Mono accumulation.

In order to monitor the accumulation of monomeric and aggregated Tau over time, iPSCNs were treated with red-labeled Mono and green-labeled S-fib for 3 h. Then, they were imaged immediately or after 21 h of incubation in differentiation media without Tau. This approach enabled us to visualize cell internalized Tau after short and prolonged incubation. After 3 h of incubation, green, red, and a few yellow puncta (containing both Mono and S-fib) were visible and distributed in the soma and neurites (Suppl. Fig S13, Additional file 1). However, after 21 h, larger yellow dots were observed, mainly localized in the soma, suggesting that internalized Tau was compiled over time and transferred to soma. Treatment of iPSCNs with Oligo Tau showed similar results (Suppl. Fig. S14, Additional file 1). Repeating this experiment in LUHMES neurons showed similar results in 3 h, while smaller and more distributed green, red, and yellow puncta appeared. Similar to iPSCNs, after 21 h, larger dots were localized mainly in the soma (Suppl. Fig S15, Additional file 1). This data suggests that, following the internalization, small puncta of eTau Monomers and aggregates are dispersed throughout the soma and neurite. However, over time, they accumulate in the soma, where they colocalize with each other in larger puncta.

## Discussion

**Fig. 8:**
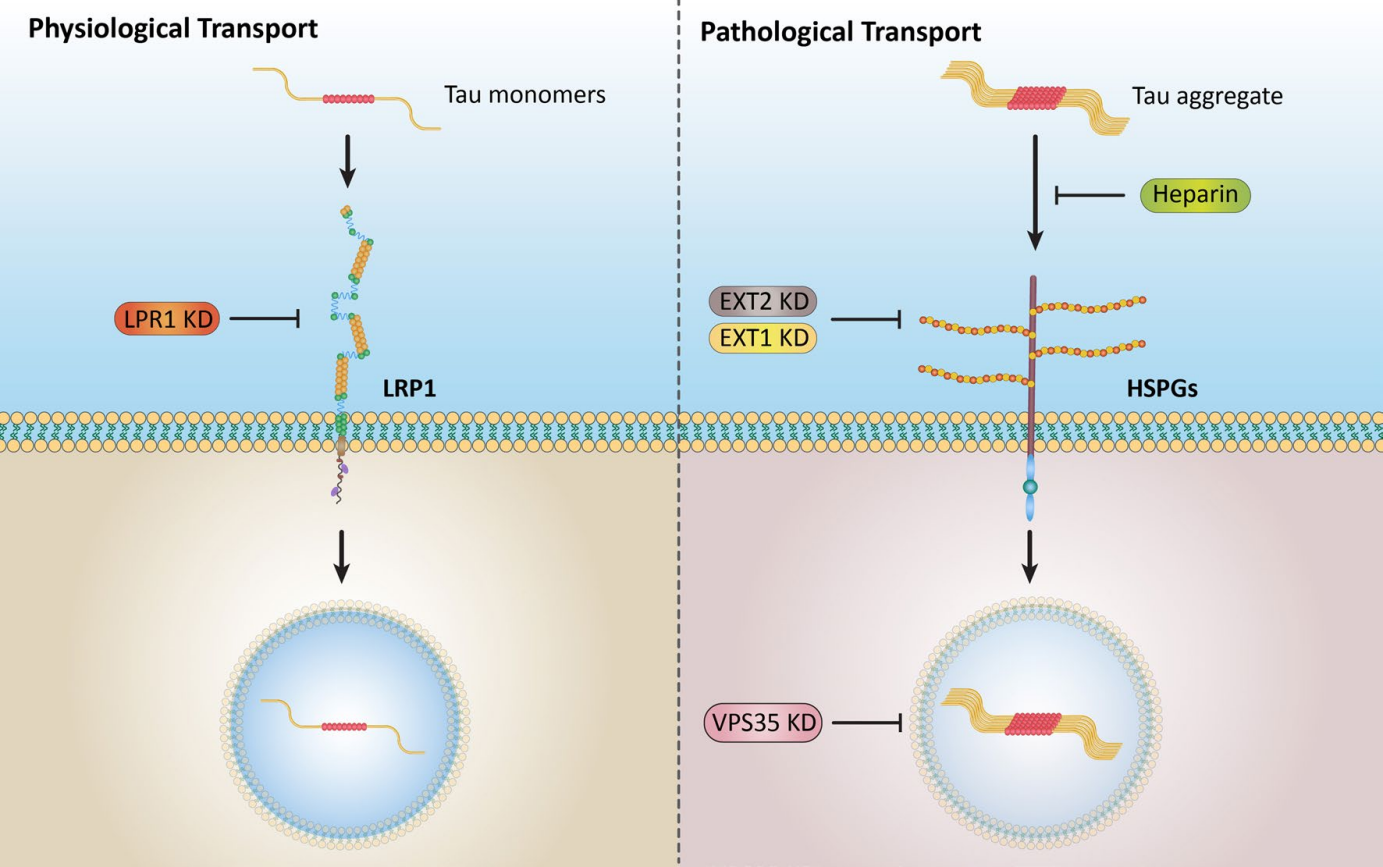
Model of differential uptake and accumulation of Tau Monomers and aggregates in human neurons. Under physiological conditions, Tau Monomers exist in the extracellular environment and internalize neurons via LRP1-mediated endocytosis, which can be inhibited by LRP1 knockdown. Under pathological conditions, Tau aggregates in the extracellular environment internalize neurons mainly via HSPGs mediated endocytosis, which can be blocked by heparin or knockdown of HSPGs synthetizing enzymes such as EXT1 and EXT2. LRP1 may be partially involved in aggregate uptake in some types of neurons, which needs further investigation. The downregulation of VPS35, as a critical component of the retromer complex, reduced the accumulation of aggregates in both models of human neurons in this study. The endocytic vesicles inside the cells are depicted with faded colors since the internalization of Tau might be via endocytic vesicles and/or direct cytosol entry, which was not investigated in this study

Here, to study Tau uptake, we generated a recombinant Tau aggregated mixture with conformational similarities to patients’ derived aggregates and fractionated it into more homogenous populations of Tau species. Biochemical and biophysical characterization confirmed the structural distinctions between the fractions. Among them, intermediate Tau aggregates, including oligomers and small fibrils, were found to be potent seeds for prion-like propagation. We used a competition assay to compare the neuronal uptake and accumulation of well-characterized seed-competent assemblies and physiological Tau Monomers. Our results indicated that extracellular Tau (eTau) aggregates and monomers have distinct uptake pathways, leading to a lower neuronal accumulation of monomers than aggregates. Evaluating various cellular and molecular mechanisms confirmed the role of specific molecular mediators, including LRP1, HSPGs, and VPS35, in the differential regulation of neuronal accumulation for physiological Tau Monomers versus pathology-relevant aggregates, as illustrated in Fig. 8.

The reactivity of the heparin-induced aggregates with conformational antibodies of MC1, TNT-1 and TOMA revealed the accumulation of pathologic epitopes in the fibrillization process and confirmed the pathology-relevance of these aggregates. Unexpectedly, the reactivity toward TOMA, as an oligomeric antibody, appeared in the late stage of fibrillization. Previously, the presence of multiple oligomeric structures in heparin-induced fibrillization revealed the possibility of forming oligomers in independent pathways during the fibril formation process [59]. Moreover, the reactivity of an anti-oligomeric antibody, A11, toward Tau fibrillar fractions has been reported [12]. Thus, this evidence suggests that oligomers or epitopes associated with them can appear in the late stage of the fibril formation process.

AFM imaging and SEC analysis revealed the presence of various Tau species with different biophysical features in the fibrillization-derived aggregates, which aligns with previous studies [12]. By using physical separation similar to a prior study on α-synuclein aggregates [60], we generated a more homogenous population of aggregates. Although a complete separation between the aggregated species is not feasible, AFM, TEM, CD, density gradient centrifugation and SEC analysis confirmed species enrichment with different sizes, densities, and structures in the fractions.

Various definitions have been introduced for Tau oligomers. They have been defined as toxic species with variable structures, ranging from dimers to multimers, granular forms, and small filamentous aggregates [61], also as soluble aggregates and the most toxic species [62], or simply as intermediate entities [63]. Although our fractionation technique can not fully eliminate contamination to small fibrillar structures, our methods enriched a wide range of intermediate structures, from dimers to multimers and oligomers. Characterization of the fractions revealed that oligomeric fractions’ size, density, and structure are intermediate between monomeric and fibrillar fractions. More importantly, DLS analysis showed a size distribution of 20–80 nm for soluble oligomeric Tau that is comparable to previous studies on Tau oligomers [12, 39]. Moreover, the oligomeric fraction was the only toxic fraction among others, which aligns with previous reports [64–66]. Thus, despite the lack of consensus on the Tau oligomers’ definition and characteristics, our data suggest that the oligomeric fraction in this study is enriched with intermediate aggregates and represent the main features of Tau oligomers that has been described before.

We verified that small fibrils and oligomers are potent seeds based on the prion-like propagation hypothesis due to a higher uptake rate, cytosol entry capacity, and seeding endogenous aggregation potential [10, 67]. Previous studies showed that fragmented (sonicated) fibrillar structures of prion, Aβ, and α-synuclein were more efficient in seeding activity than large (non-sonicated) fibrils [41, 68, 69]. We did not find differences between Tau aggregated species in seeding activity using our endogenous aggregation assay. However, our result confirmed the lower efficiency of large fibrils in intracellular accumulation. Thus, our primary focus here was studying the uptake and accumulation of Tau oligomers and small fibrils as potent spreading species due to high seeding activity and high intracellular accumulation efficiency.

Our data showed a higher neuronal accumulation of Tau aggregated mixture compared to physiological monomers, suggesting the difference in the transport mechanism between the Tau species. To ensure that the higher intensity of aggregates is not associated with a higher fluorescence of labels, the labels’ intensity was measured in aggregates status and after unfolding to monomers. The results revealed that in the aggregated state, a quenching effect reduces the intensity of labels on aggregates. Thus, the labels on aggregates are even less bright than those on monomers. Moreover, measuring the total fluorescence intensity by plate reader enabled us to avoid any interference related to the distribution of fluorescent labels in cells between monomers and aggregates.

Using a novel competition assay, we aimed to examine whether the uptake and intracellular accumulation of eTau Monomers and intermediate aggregates are distinct or overlapping. Our results showed a partial cross-species competition in iPSCNs and no competition in LUHMES neurons, further supporting the differences in the neuronal accumulation pathway of eTau Monomers and aggregates. Higher uptake of Tau aggregates compared to monomers has been reported in C17.2 mouse neuronal precursor cells [15] and primary mouse neurons [70]. Contrasting results were obtained from studying HEK293T cells [71], possibly due to using a proliferating cell with a dynamic membrane compared to non-diving neuronal cells. This highlights the considerable variation among cells for the cellular uptake mechanisms of Tau.

A study using a pH-sensitive tag suggested a similar uptake rate between Tau Monomers and fibrils during 3 h in iPSCNs [72]. While their result reflects only low-pH vesicle Tau accumulation, not total accumulation, our kinetic study revealed a similar total accumulation rate of monomers and aggregates within the first 3 h of exposure. A significantly higher accumulation of aggregates appears after 3 h of exposure, suggesting the role of a post-internalization process in the differential neuronal accumulation of Tau species. This might be related to the differential recovery rate of receptors. To test this, we pretreated cells with either unlabeled monomers or aggregates and then exposed them to fluorescently labeled Tau of the same species. Our result showed no impact on monomer accumulation, while the accumulation rate of aggregates reduced significantly, confirming the saturation of aggregates’ internalization mechanism but not monomers. This might be explained by the different receptor recovery rates between monomers and aggregates. The binding of Tau aggregates might be stronger to their respective receptor, especially due to high surface adherence. Moreover, this might be associated with the dysfunction of vesicle sorting and the endosomal system, as Tau aggregates were shown to impair autophagic flux by dysregulating the ESCRT-III complex as part of vesicle sorting machinery [73]. Additionally, it has been shown that the inhibition of amyloid deposition rescues the autophagy-lysosomal pathway dysfunction [74].

HSPGs have been found to be the first molecular mediator of Tau cellular internalization [16]. However, a subsequent study proposed that the uptake of Tau is independent of glycosaminoglycans [75]. The contradictory results could be rooted in using various Tau species; the first study used Tau fibrils, while the second used Tau Monomers. Here, we tested the role of HSPGs in Tau uptake by using heparin as a molecular inhibitor of HSPGs and by siRNA-mediated knockdown of genes involved in HSPGs’ synthesis, including EXT1 and EXT2. Our data confirmed that in iPSCNs and LUHMES neurons, HSPGs are critical for the uptake and accumulation of Tau aggregates but not monomers. Despite the consensus on the role of HSPGs in the uptake of Tau aggregates [76], there are discrepancies in the role of HSPGs in the uptake of Tau Monomers. The uptake of Tau Monomers was reported to be HSPGs-independent in astrocytes [77], while the knockout of EXT2 was shown to reduce the uptake of Tau Monomers intensively in H4 neuroglioma cells and slightly in iPSCNs [17]. The HSPGs-dependent uptake of Tau Monomers has been identified in C6 glioma cells [78]. These contradictory results might be explained by the cell type differences in the case of C6 glioma cells, methodological differences in the case of iPSCNs, and both parameters in the case of H4 neuroglioma cells. Further studies would be necessary to conclude the role of HSPGs in the uptake of Tau Monomers in human neurons.

A recent study discovered LRP1 as a critical receptor for Tau [19]. They showed that the knockout of LRP1 completely blocked the uptake of Tau Monomers in neuroglioma cells and iPSCNs but only partially reduced the uptake of sonicated fibrils in neuroglioma cells. Similarly, a CRISPR screen for Tau uptake in iPSCNs reported LRP1 as one of the top-ranked genes for the uptake of monomers but not for oligomers [79]. Here, we tested the impact of LRP1 knockdown in human neurons and found that the uptake of monomers was intensively dependent on LRP1 in both neuronal types. However, the uptake of Tau aggregates was partially dependent on LRP1 in iPSCNs and entirely independent of LRP1 in LUHMES neurons. Moreover, we showed that LUHMES neurons with a very low expression of LRP1 can still accumulate Tau aggregates at a similar rate to iPSCNs with high LRP1 expression. This suggests that LRP1 may assist the uptake of Tau aggregates, but it is not critical for this process. As we showed with siRNAs, the knockout of LRP1 in CHO cells also confirmed that LRP1 is not the sole receptor for Tau [80], and there are other uptake mechanisms in human neurons for Tau aggregates independent of LRP1. Thus, further research would be crucial to elucidate the role of LRP1 in the uptake of Tau aggregates, especially in human neurons.

The Tau oligomers’ uptake was reported to be highly dependent on LRP1, similar to monomeric Tau [19]. However, our results suggest that the uptake of Tau oligomers is comparable to Tau fibrils and has partial or no dependency on LRP1 in iPSCNs and LUHMES neurons, respectively. This controversy is probably due to the differences in the preparation protocol of Tau oligomers. Rauch and colleagues induced oligomerization via a 4-hour incubation protocol, and no purification or enrichment was specified. As the yield of oligomer production is typically low, lack of enrichment may cause contamination with a pool of monomers that can justify the similarity of results with Tau Monomers. Here, we enriched the oligomers by removing the monomers using ultrafiltration to obtain a more homogenous population of soluble aggregates. SEC analysis and immunoassay confirmed the lack of monomer contamination in the oligomeric fraction. This highlights the importance of thorough characterizations for interpreting any results associated with the intermediate aggregated species.

Despite many similarities, we found differences between the human neuronal models we used in this study, especially regarding the role of LRP1 in Tau uptake. First, we found that the uptake of Tau aggregates was partially dependent on LRP1 in iPSCNs, while it was mainly independent of LRP1 in LUHMES neurons. Second, the cellular accumulation of Tau Monomers, which is substantially reliant on LRP1, was markedly higher in iPSCNs compared to LUHMES neurons. Our western blot analysis revealed a much higher expression level of LRP1 in iPSCNs compared to LUHMES neurons, which explains these variations. There are two main distinctions between the neuronal models used in this study. The first distinction is the neuronal type since iPSCNs in this study represent the cortical lineage with secondary differentiation [33], while LUHMES neurons are derived from primary midbrain tissue and represent dopaminergic neurons [81]. Second, the degree of neuronal maturity varies between these cells; iPSCs underwent an epigenetic reset to the pluripotent state of human embryonic stem cells [82], while LUHMES neurons were developed for eight weeks in vivo before isolation [83]. Various studies showed that LUHMES neurons differentiate into mature and functional neurons within one week [84, 85]. In contrast, variation in the maturity degree has been reported for neurons derived from iPSCs [86, 87]. The first study that reported the role of LRP1 on the uptake of Tau aggregates was carried out on iPSCNs with a short differentiation of 14 to 18 days [19]. However, a CRISPR screen that did not find LRP1 within the threshold range for aggregate uptake was performed on iPSCNs with long differentiation of 65 days [79]. Altogether, these pieces of evidence highlight the importance of further studies on the role of LRP1 in the uptake of Tau aggregates in various types of human neurons with various degrees of maturity.

VPS35 is an essential component of the retromer complex [88], which is involved in endosomal transmembrane protein recycling and cargo cell sorting. The contribution of VPS35 to several neurodegenerative diseases has been reported before [89]. Here, using two different models of human neurons, we showed that VPS35 knockdown reduced the accumulation of aggregated Tau in both iPSCNs and LUHMES but increased the accumulation of Tau Monomers in LUHMES. Consistent with this bidirectional dependence, the knockdown of VPS35 in HEK cells increased the cytosol entry of Tau aggregates [37]. Despite the different impacts, this report also confirms the role of VPS35 in the trafficking of extracellular Tau. The effect of VPS35’s knockdown on Tau Monomers’ accumulation differed from aggregates in both neuronal types, suggesting differences in intracellular sorting of Tau Monomers and aggregates. Further research would be necessary to verify the role of VPS35 and retromer complexes in Tau transport. Moreover, additional studies and genetic screens are essential to confirm the role of vesicle sorting machinery in Tau transport and to develop a comprehensive picture of regulators for differential neuronal accumulation of physiological and pathological Tau species.

Our results indicate that the intracellular accumulation of Tau aggregates is mainly HSPG-mediated and endocytosis-dependent in human neurons. However, recent studies showed a direct HSPG-mediated translocation of Tau aggregates into cytosol independent of the endocytic pathways, which may play a critical role in seeding endogenous Tau [37, 90, 91]. Further investigation would be necessary to address the endolysosomal system’s role in Tau’s prion-like propagation.

Normal monomeric and abnormal aggregated Tau can be secreted to the extracellular environment via different physiological and pathological processes [92, 93]. The eTau has been identified in the synaptic vesicles [23], exosomes [94–96], and ectosomes [97]. However, the later study quantified 90% of eTau as vesicle-free Tau, confirming the previous studies that reported the vesicle-free Tau as the main extracellular form [98–102]. An unconventional secretion associated with HSPGs has been found for pathology-associated vesicle-free Tau [103, 104]. Therefore, we mainly focused on vesicle-free eTau uptake here. However, whether Tau seeds propagate in a vesicle-free or vesicle-associated format in the pathologic brain is still unclear. Further research on the cell-to-cell transport and seeded aggregation of vesicle-associated eTau species would be necessary to provide greater insight into the spread of Tau pathology.

## Conclusions

In summary, we identified intermediate Tau aggregates, including oligomers and small fibrils, as potent seeds for prion-like propagation of Tau pathology. Moreover, we found that the neuronal uptake and accumulation of seed-competent pathology-relevant aggregates were differentially regulated from physiological monomers. Our gene knockdown experiment in human neurons revealed that monomers’ uptake is mainly dependent on LRP1, while aggregates’ uptake primarily depends on HSPGs. Moreover, the downregulation of VPS35 as a component of vesicle sorting machinery differentially modulates the cellular accumulation of Tau Monomers and aggregates. These findings shed light on the possibility of targeting pathological Tau spreading without disturbing the probable physiological intercellular transport of native monomeric, i.e., non-pathogenic Tau, for developing future therapeutic strategies.

## Electronic supplementary material

Below is the link to the electronic supplementary material.

Supplementary Material 1

## Data Availability

Raw data is available from the corresponding authors upon reasonable request.

## Abbreviations

AFM: Atomic Force Microscopy
BSA: Bovine Serum Albumin
CD: Circular Dichroism
CME: Clathrin-Mediated Endocytosis
cof-free-fib: co-factor-free fibrils
eTau: extracellular Tau
EXT1: Exostosin Glycosyltransferase 1
F-mono: fibrillization-derived monomeric fraction
FL: fluorescently labeled
HSPGs: Heparan sulfate proteoglycans
iPSCNs: induced pluripotent stem cells derived neurons
L-fib: large fibrils fraction
LRP1: low-density lipoprotein receptor-related protein 1
LUHMES: Lund human mesencephalic
Mono: monomeric fraction
Oligo: oligomeric fraction
S-fib: small fibrils fraction
SEC: Size Exclusion Chromatography
TEM: Transmission Electron Microscopy
ThT: thioflavin-T
VPS35: Vacuolar protein sorting-associated protein 35
