# Supplementary material for: Distinct regulation of Tau Monomer and aggregate uptake and intracellular accumulation in human neurons
Source: Mol Neurodegener. 2024 Dec 31;19:100. doi: 10.1186/s13024-024-00786-w (PMC11686972; doi:10.1186/s13024-024-00786-w)
Supplement: Supplementary file 1 — Supplementary Material 1 [file 13024_2024_786_MOESM1_ESM.docx]

**Additional file 1 - Supplementary information**

**A**

**B**

**Supplementary Figure S1** The effect of disaggregation with Guanidine Hydrochloride on the fluorescence of ATTO-488 in Tau monomers and aggregates. **A** Thioflavin T (ThT) assay for unlabeled Tau aggregates in the untreated control (UTC) and after treatment with 2M Guanidine Hydrochloride (GuHCl). **B** Fluorescence intensity of ATTO-488 of labeled Tau monomers and aggregates in UTC and after treatment with 2M GuHCl. Error bars represent SD. One-way ANOVA followed by post-hoc test **p<0.01, ***p<0.001, ****p<0.0001 versus UTC.

**A**

**B**

BSA Mono

Without quencher


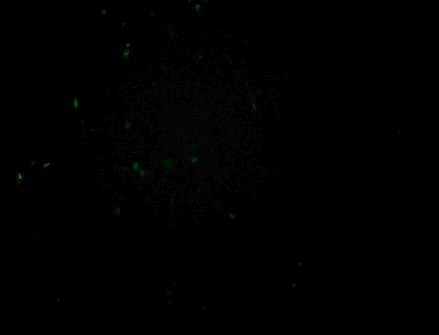

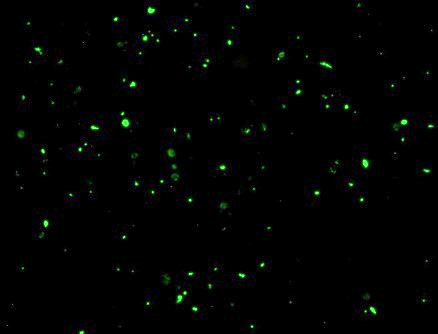

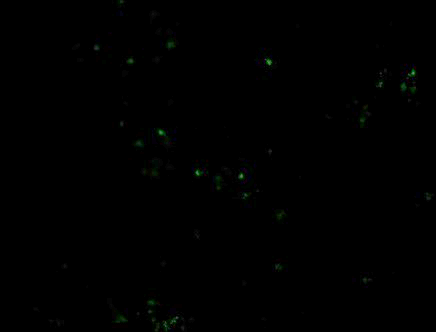

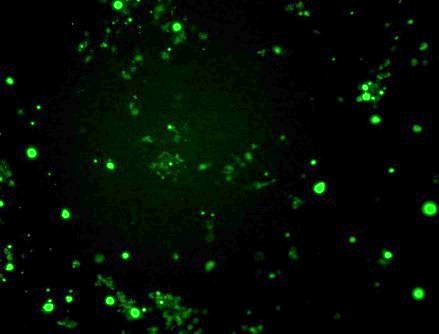


With quencher

BSA Agg

**Supplementary Figure S2** Bovine serum albumin (BSA) monomers and amyloid aggregates do not accumulate in iPSC-derived neurons. **A** Proteostat fluorescence measurement of 4% BSA-CF488A before and after 24 hours of fibrillization. **B** Fluorescence images of cells treated with 100 nM 4% BSA-CF488A monomers (Mono) and aggregates (Agg) after 24 hours in the absence and presence of the quencher. Error bars represent SD. n=4 per experimental condition. One-way ANOVA followed by post-hoc test ****p<0.0001.

5% Iodixanol 50%

**Supplementary Figure S3** Iodixanol gradient centrifugation for soluble and insoluble Tau fractions. Fluorescence measurements of fluorescently labeled fractions of large fibrils (L-fib), small fibrils (S-fib), and soluble fraction (Soluble F.) of Tau aggregation after separation in a 5% to 50% iodixanol gradient columns and fractionation.

**C**

**D**

**B**

**A**

**Supplementary Figure S4** Comparing the conformational differences between labeled and unlabeled Tau monomers and aggregates. Circular dichroism graphs of labeled and unlabeled Tau **A** Large fibrils (L-fib), **B** small fibrils (S-fib), **C** oligomers (Oligo), and **D** fibrillization derived monomer (F-mono). mdeg: millidegrees.

**Supplementary Table S1.** Labeling efficiency of Tau fractions.

| **No.** | **Fractions** | **Average degree of labeling (dye-to-protein molar ratio)** |
| --- | --- | --- |
| **1** | Large fibrils | 0.417 |
| **2** | Small fibrils | 0.466 |
| **3** | Oligomers | 0.428 |
| **4** | Fibrillization-derived monomers | 0.421 |

`

**B**

**A**

**D**

**C**

**Supplementary Figure S5** Intracellular accumulation of Tau in differentiated LUHMES neurons. **A** Kinetics of intracellular Tau accumulation during 48 hours in LUHMES neurons exposed to 100 nM Tau fraction including large fibrils (L-fib), small fibrils (S-fib), oligomers (Oligo) and fibrillization-derived monomers (F-mono). **B** Titration of intracellular Tau accumulation in LUHMES neurons after 20 hours of exposure to different concentrations of Tau fractions (12.5 – 250 nM). Comparison between the kinetics of intracellular accumulation in iPSC-derived neurons (iPSCNs) or LUHMES neurons for 100 nM **C** fluorescently labeled monomers (FL-Mono) and **D** fluorescently labeled small fibrils (FL-S-fib). The monomers' labeling degree in this experiment is about ten times higher than S-fib. Error bars represent SD; n=3 per experimental condition. One-way ANOVA followed by post-hoc test; ****p<0.0001, **p<0.01, *p<0.05.


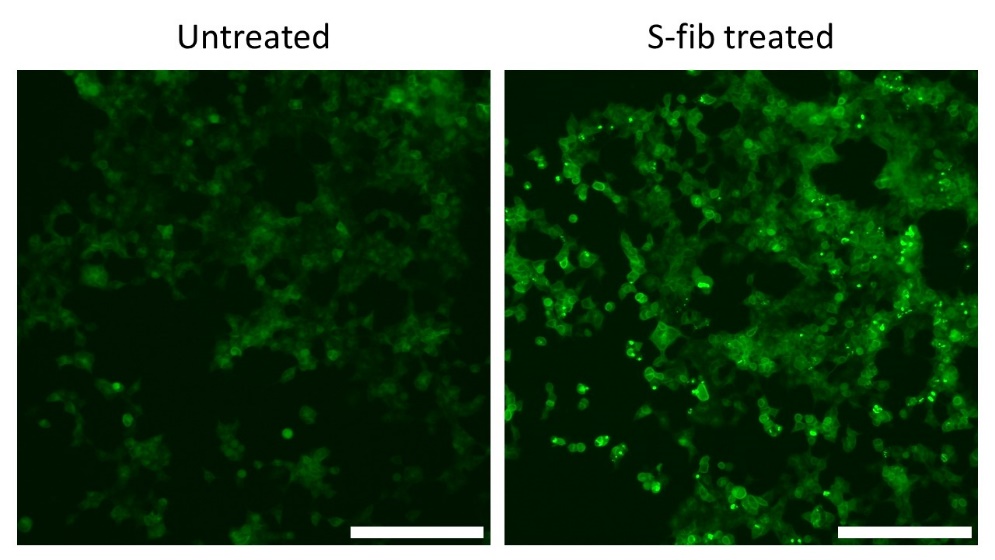


**Supplementary Figure S6** Endogenous aggregation assay. Fluorescence microscopic images of HEK293-biosensor cells expressing P301S Tau-venus. Left, untreated, and right treated with 200 nM unlabeled Tau small fibrils (S-fib). Scale bar: 250 μm.


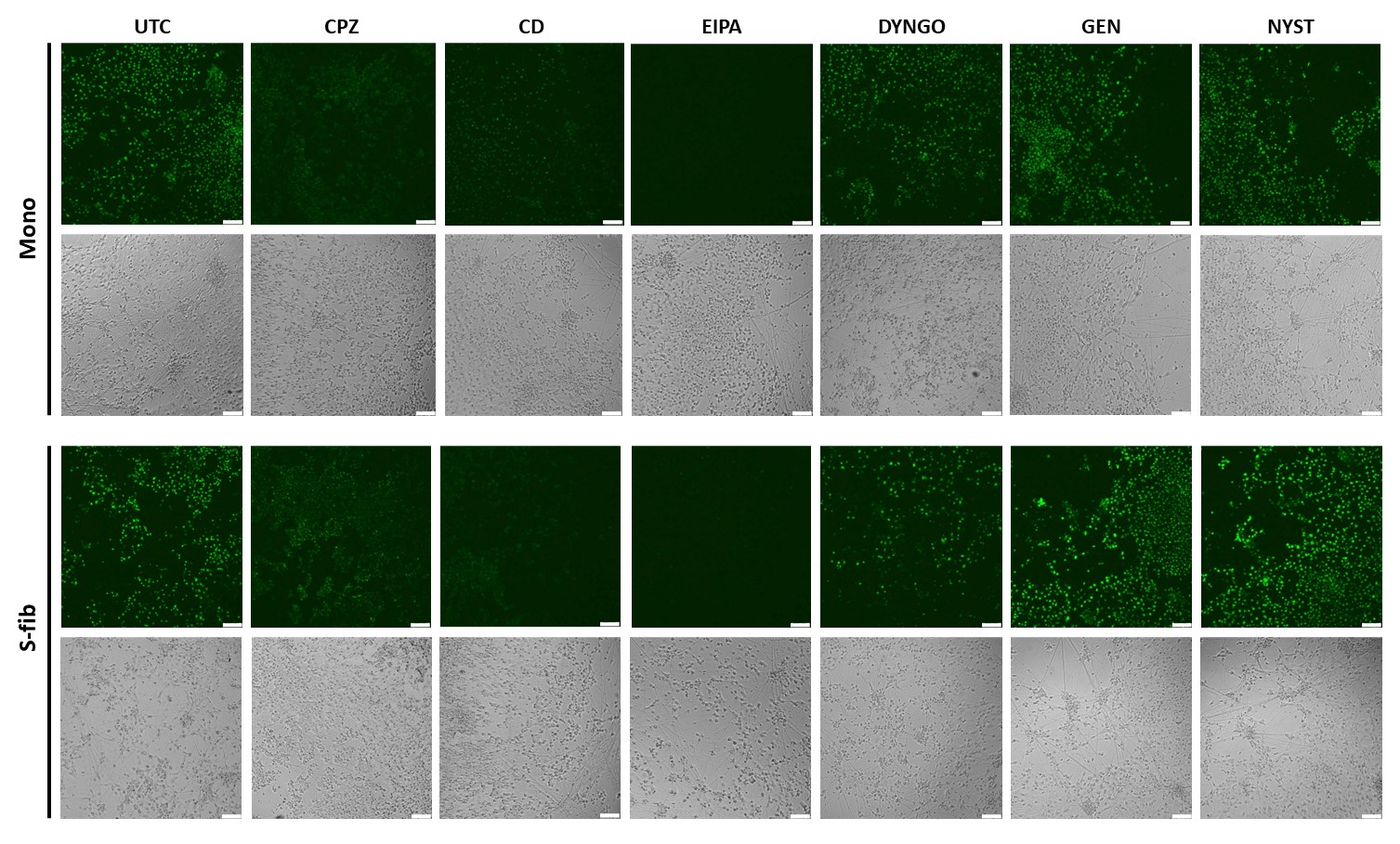


**B**

**A**

**
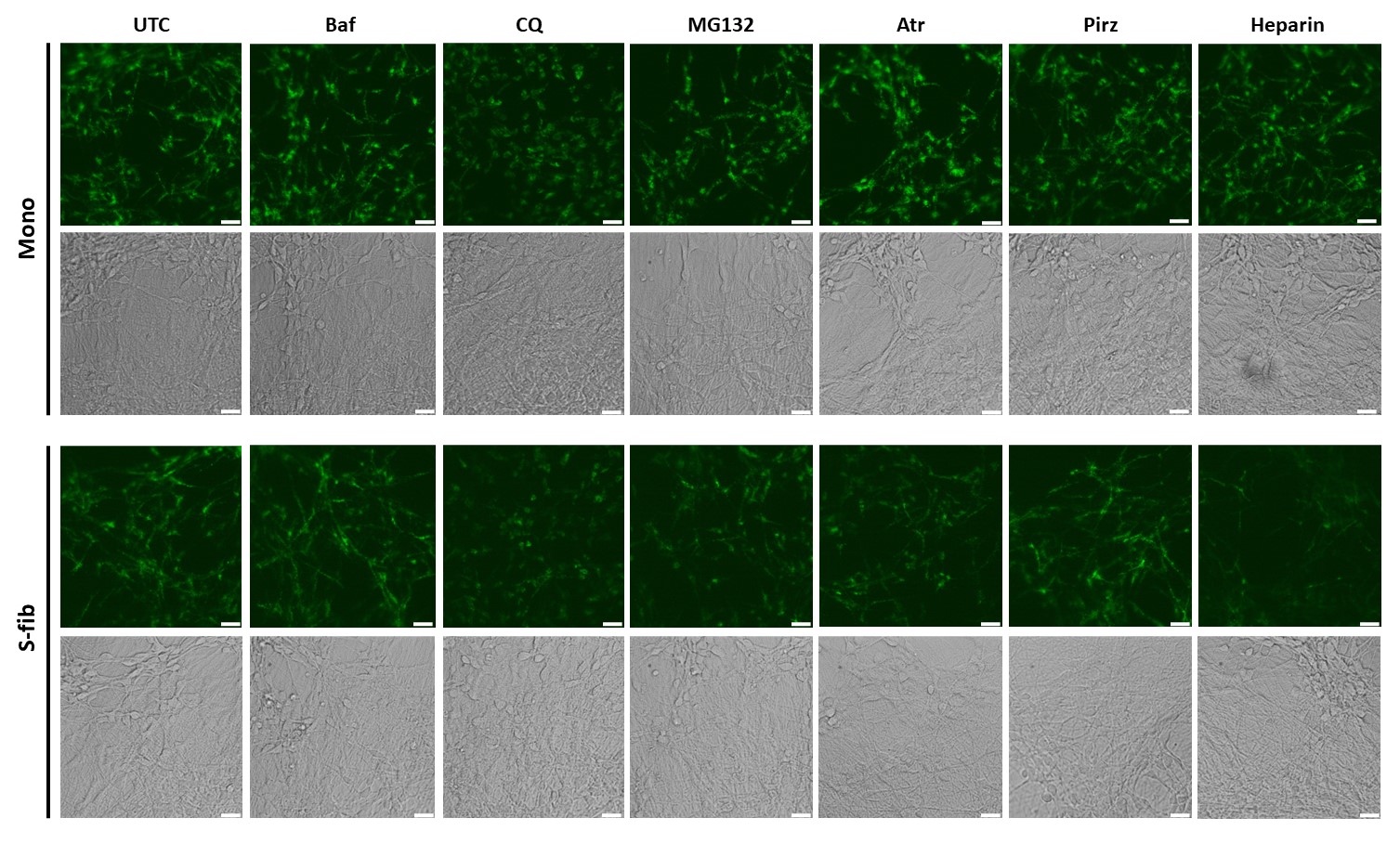
**

**Supplementary Figure S7** Representative images of Tau uptake in iPSCNs treated with small molecule inhibitors. **A** Intracellular Tau accumulation in cells left untreated as control (UTC) or treated with 50 μM Chlorpromazine (CPZ), 20 μM Cytochalasin D (CD), 30 μM 5-N-ethyl-N-isopropyl amiloride (EIPA), 75 μM Dyngo-4a (DYNGO), 200 μM Genistein (GEN), or 10 μM Nystatin (NYST) for 30 min before incubation with fluorescently labeled monomers (FL-Mono), and small fibrils (FL-S-fib), both at 250nM concentration for 3 hours (exceptionally, EIPA were present during the incubation with Tau). Scale bar: 100 μm **B** Intracellular Tau accumulation in cells treated with 25 nM fluorescently labeled Tau FL-Mono, and FL-S-fib in the presence of 100 nM bafilomycin A1 (Baf), 30 μM chloroquine (CQ), 100 nM MG132, 200 μM Atropine (Atr), 20 μM Pirenzepine (Pirz) or 2 μM Heparin for 20 hours. Scale bar: 25 μm.


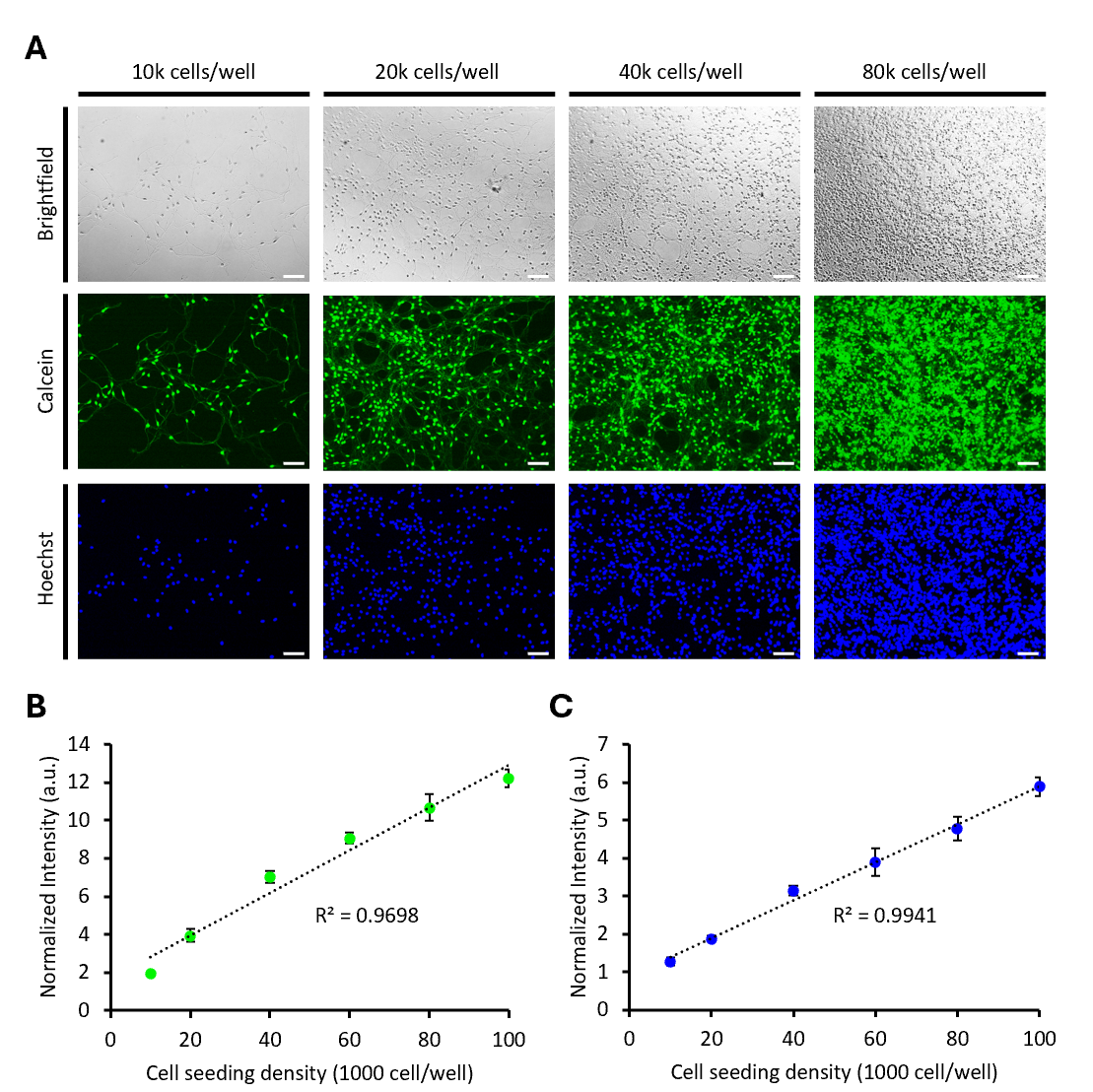


**F**

**E**

**D**


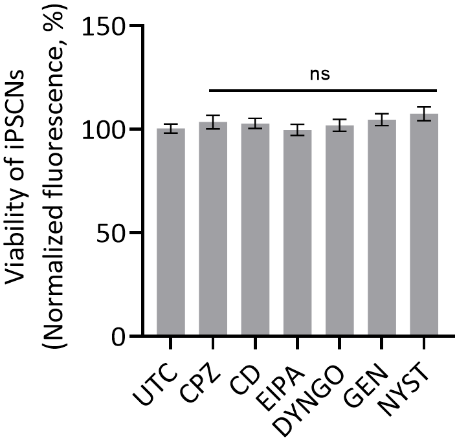

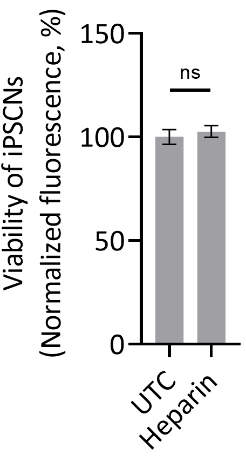

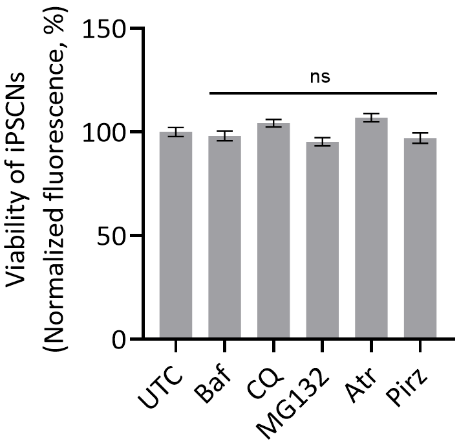


**Supplementary Figure S8** Viability assay validation and assessments. **A** Representative images of neurons seeded at different densities and stained with Calcein and Hoechst. **B** Normalized intracellular Calcein intensity measured by plate-reader from neurons seeded at different density fitted with linear trendline. **C** Normalized Hoechst intensity measured by plate-reader from neurons seeded at different density fitted with linear trendline. **D** The Calcein viability of iPSCNs treated as mentioned in Fig. S7A. **E** The Calcein viability of iPSCNs after treatment as mentioned in Fig. S7B. **F** The viability of iPSCNs treated with 2 μM Heparin for 20 hours. One-way ANOVA ns: none significant. UTC: untreated control (DMSO or water).

**B**

**A**

**Supplementary Figure S9** Effect of Tau pretreatment on the intracellular accumulation of Tau in LUHMES neurons. A Kinetics of intracellular Tau accumulation for cells pre-treated with 200 µM unlabeled Mono for 2 hours before exposure to 250 nM FL-Mono. B Kinetics of intracellular Tau accumulation for cells pre-treated with 200 nM unlabeled S-fib for 2 hours before exposure to 150 nM FL-S-fib. The significance was calculated between "No pretreat" and "Pretreat" at each time point (Only significant points were shown). Error bars represent SD. n=3 per experimental condition. One-way ANOVA followed by posthoc test; *p<0.05, **p<0.01, ***p<0.001 vs. "No pretreatment".


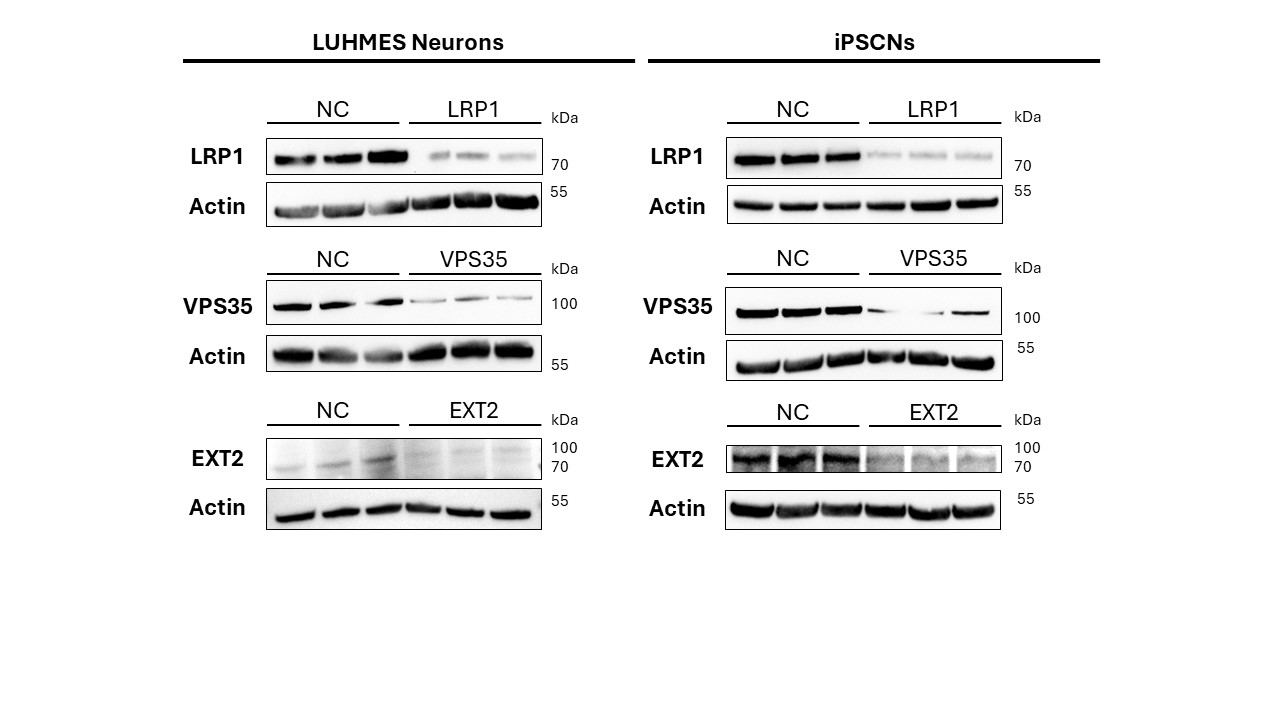

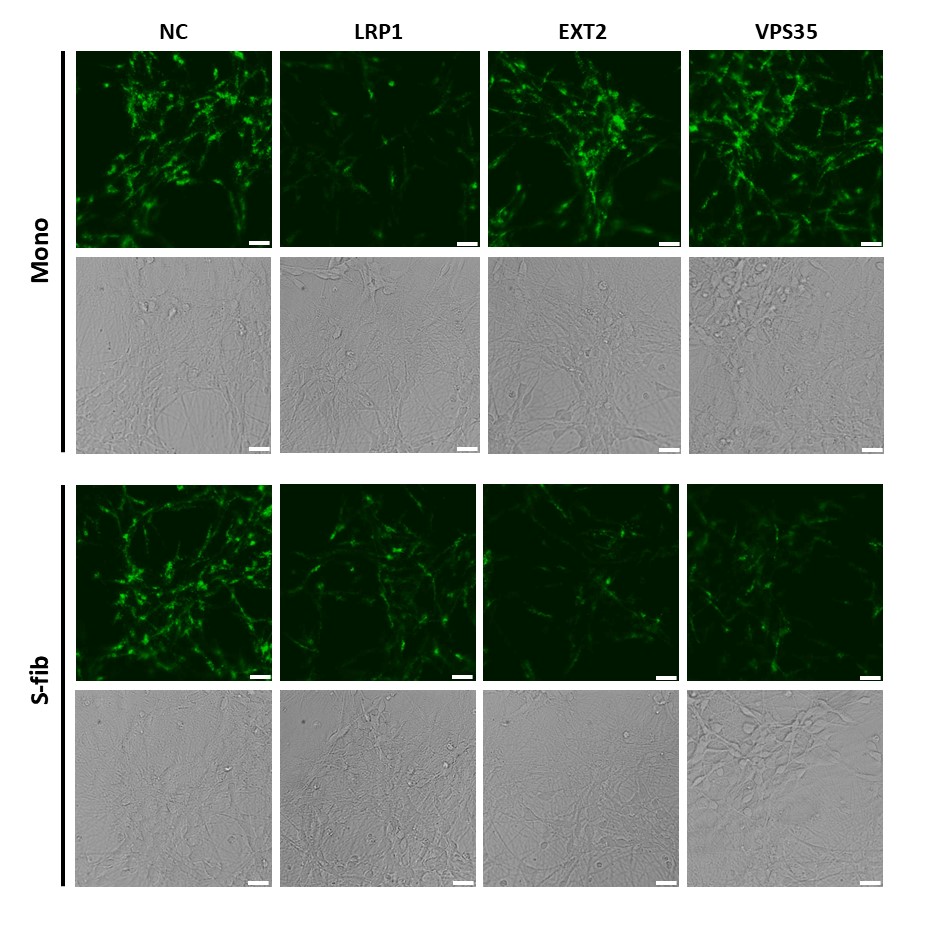


**B**

**A**

**D**

**C**


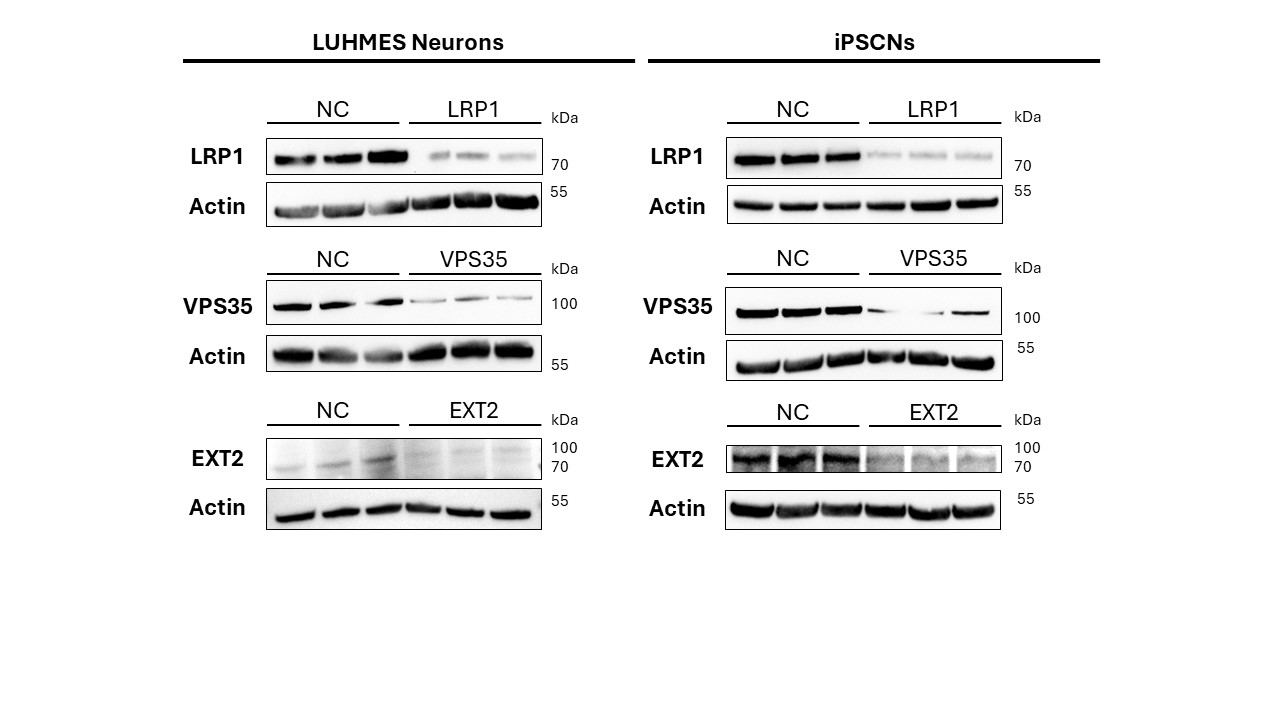

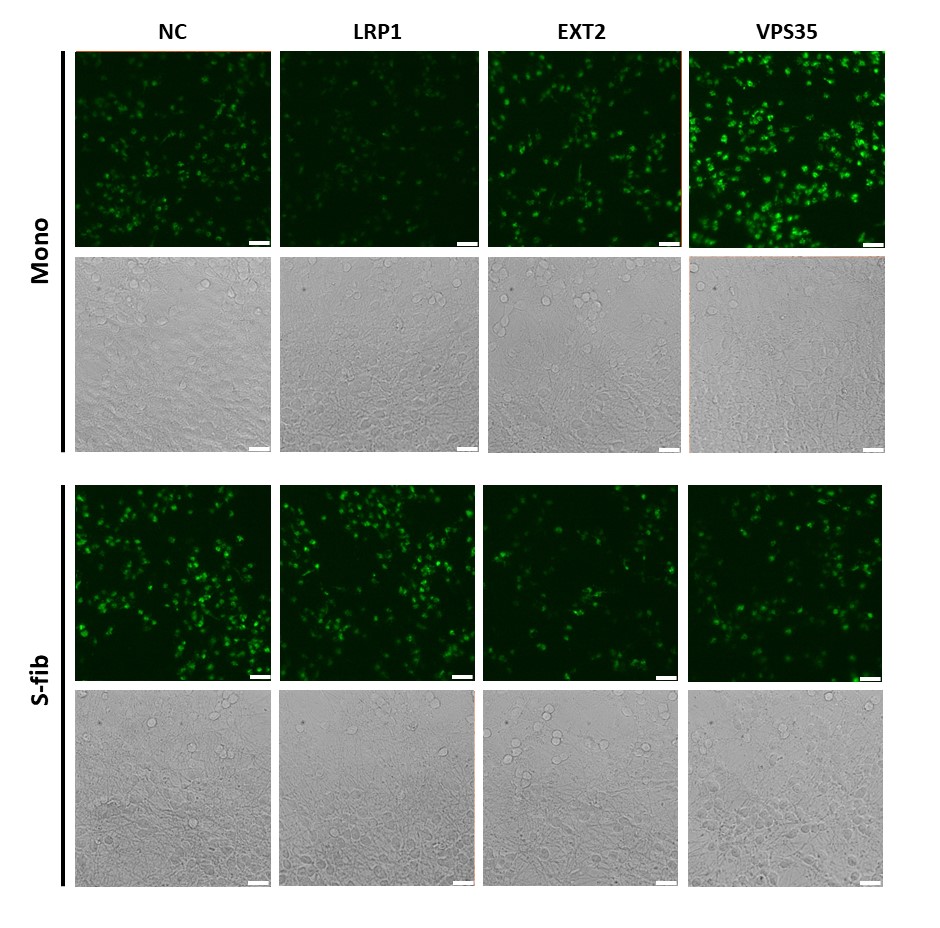


**F**

**E**


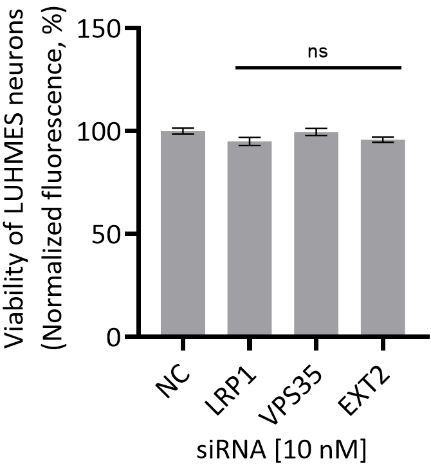

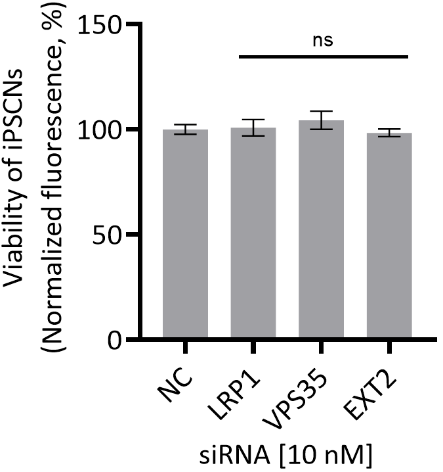


**Supplementary Figure S10** Characterization of siRNA knockdown effect in iPSCNs and LUHMES neurons. Western blot image of **A** iPSCNs and **C** LUHMES neurons treated with 10 nM siRNA. Representative images of Tau monomers (Mono) and small fibrils (S-fib) uptake in **B** iPSCNs and **D** LUHMES neurons treated with 10 nM siRNAs. Scale bar: 25 μm. **E** The viability of iPSCNs treated with 10 nM siRNAs of LRP1, VPS35 and EXT2 compared to negative control (NC). **F** The viability of LUHMES neurons treated with 10 nM siRNAs of LRP1, VPS35 and EXT2 compared to NC. One-way ANOVA ns: none significant.


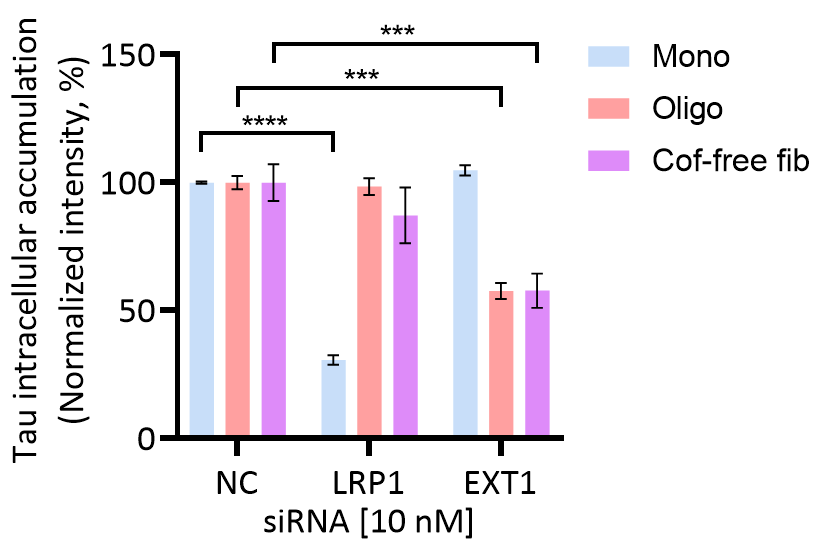

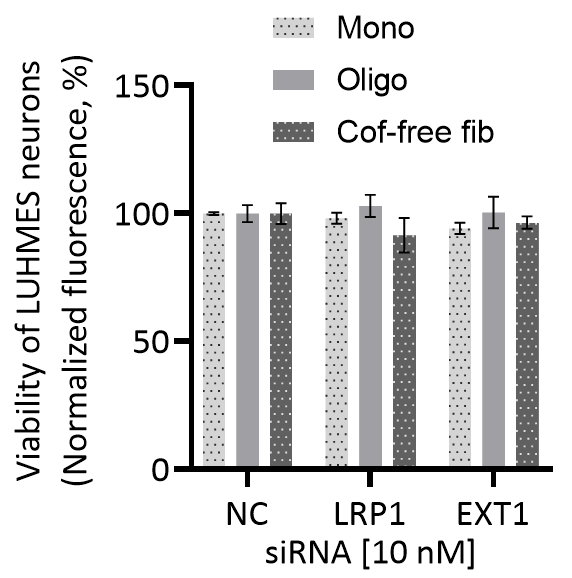


**B**

**A**

**C**

**E**

**D**


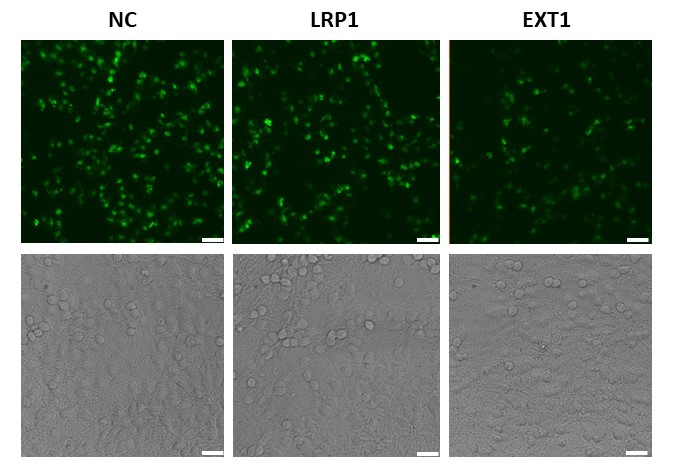


***

**Supplementary Figure S11** The impact of siRNA treatment on the intracellular accumulation of various Tau species. **A** The impact of gene knockdown on the uptake and accumulation of Tau monomers (Mono), oligomers (Oligo), and co-factor-free fibrils (Cof-free fib) in LUHMES neurons. **B** The viability of LUHMES neurons after treatment with siRNAs for non-coding (NC), LRP1, and EXT1 siRNAs. Error bars represent SEM; n=3 per experimental condition. **C** The impact of gene knockdown on the uptake and accumulation of a mixture of Tau aggregates fractions, including large fibril, small fibril and oligomer fraction. **D** The viability of LUHMES neurons after treatment with siRNAs of NC, LRP1 and EXT1. Error bars represent SEM; n=3 per experimental condition. One-way ANOVA followed by post-hoc test; ***p<0.001, ****p<0.0001, where significance is not mentioned means not significant. **E** Representative fluorescence images of C (up) with the brightfield image of the same area (down). Scale bar: 25 nm.

**B**

**A**


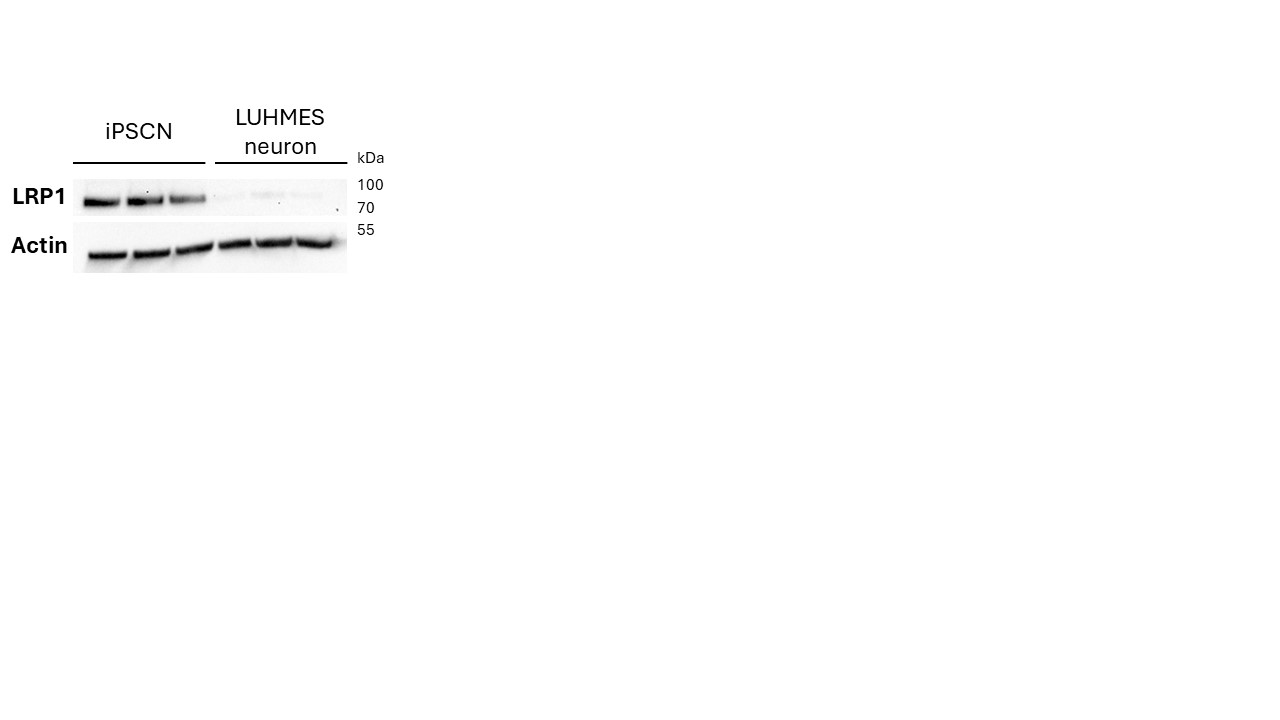


**Supplementary Figure S12** LRP1 protein level in iPSCNs and LUHMES neurons. **A** Western blot analysis of the LRP1 expression in iPSCNs versus LUHMES neurons. **B** Quantification of the western blot bands in A. Error bars represent SEM; n=3 per experimental condition. One-way ANOVA followed by post-hoc test; **p<0.01.

**A**


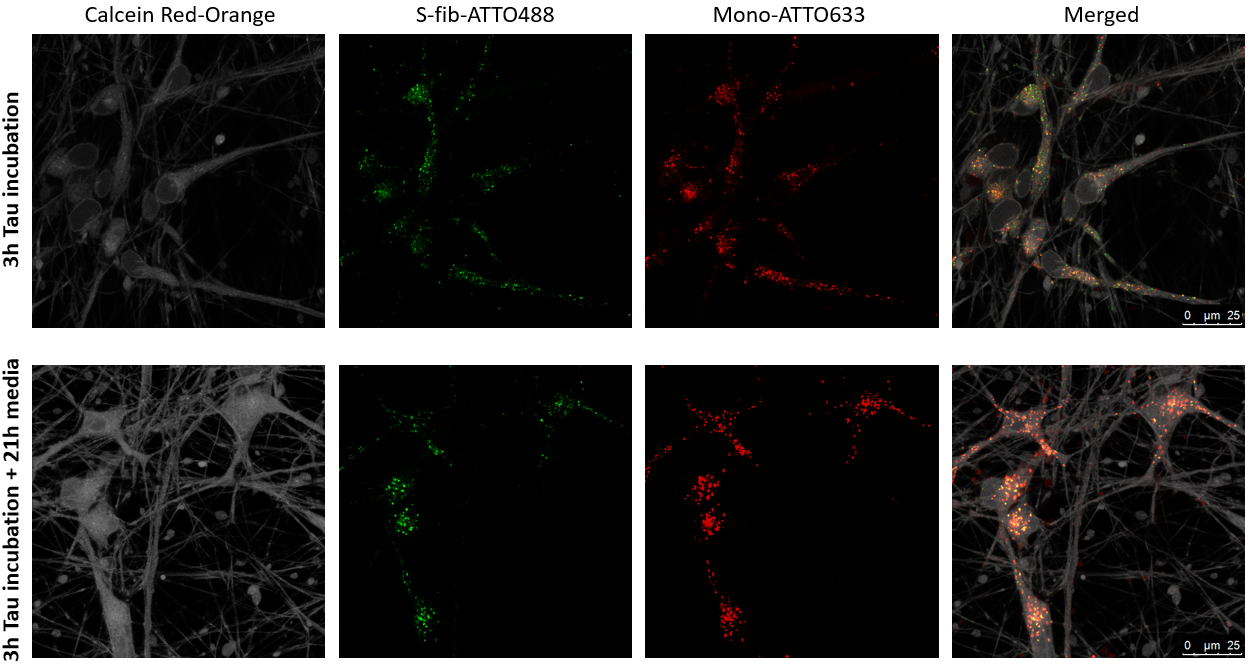

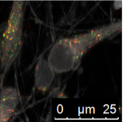

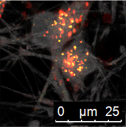


**B**


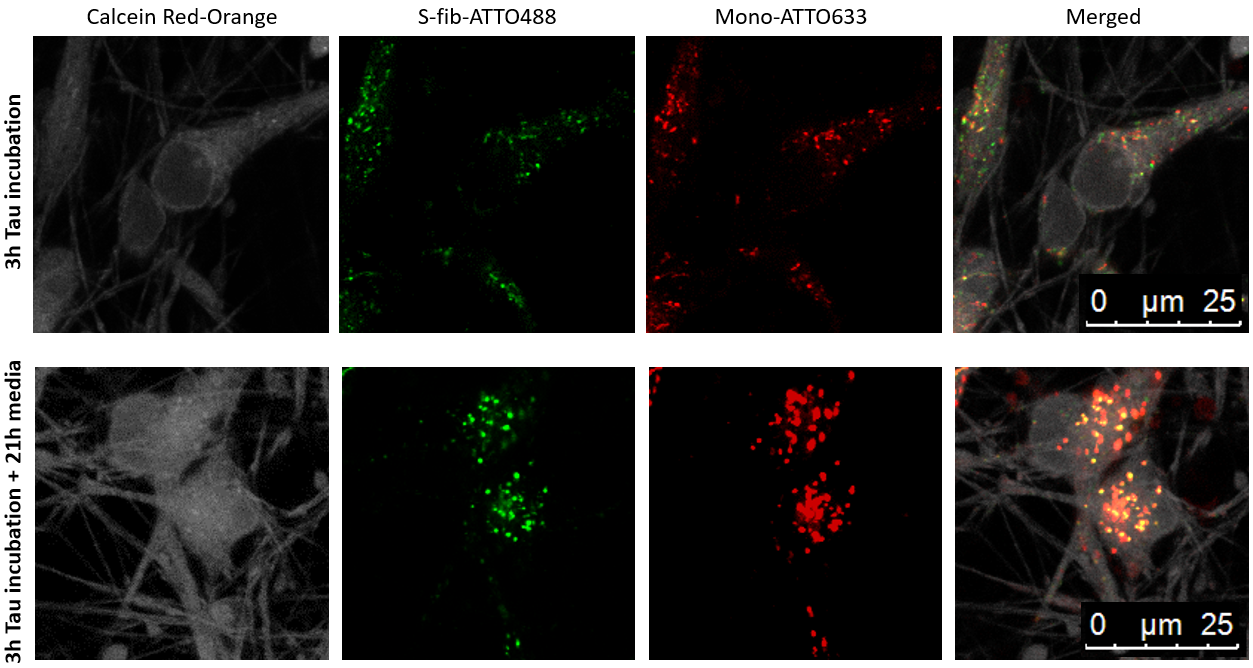


**Supplementary Figure S13** Confocal microscopy of cellular localization of Tau monomers and small fibrils after short and long incubation in iPSC-derived neurons (iPSCNs). **A** Confocal imaging of iPSCNs co-treated with 100 nM Tau monomers labeled with ATTO633 (Mono-ATTO633) and 100 nM small fibrils labeled with ATTO488 (S-fib-ATTO488). Cells were incubated with Tau for 3 hours, and imaging was performed immediately or after 21 hours of incubation in the standard differentiation media without Tau. Calcein red-orange was used as a cell tracker. **B** Higher magnification of the square in part A.

**A**


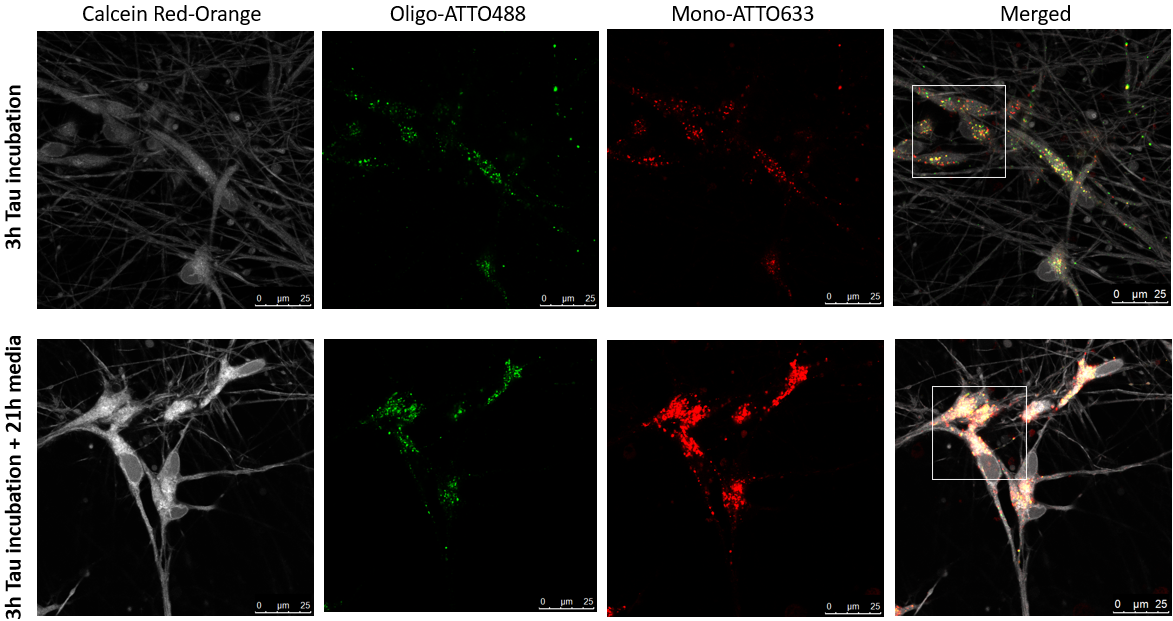


**B**


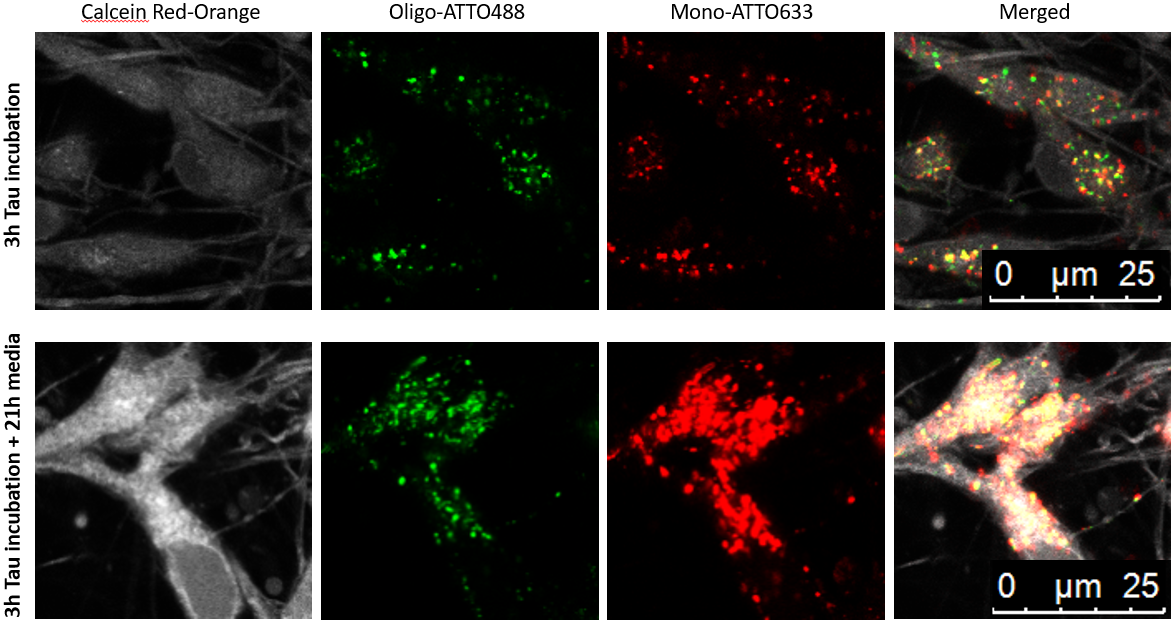


**Supplementary Figure S14** Confocal microscopy of cellular localization of Tau monomers and oligomers after short and long incubation in iPSC-derived neurons (iPSCNs). **A** Confocal imaging of iPSCNs co-treated with 100 nM Tau monomers labeled with ATTO633 (Mono-ATTO633) and 100 nM oligomers labeled with ATTO488 (Oligo-ATTO488). Cells were incubated with Tau for 3 hours, and imaging was performed immediately or after 21 hours of incubation in the standard differentiation media without Tau. Calcein red-orange was used as a cell tracker. **B** Higher magnification of the square in part A.

**A**


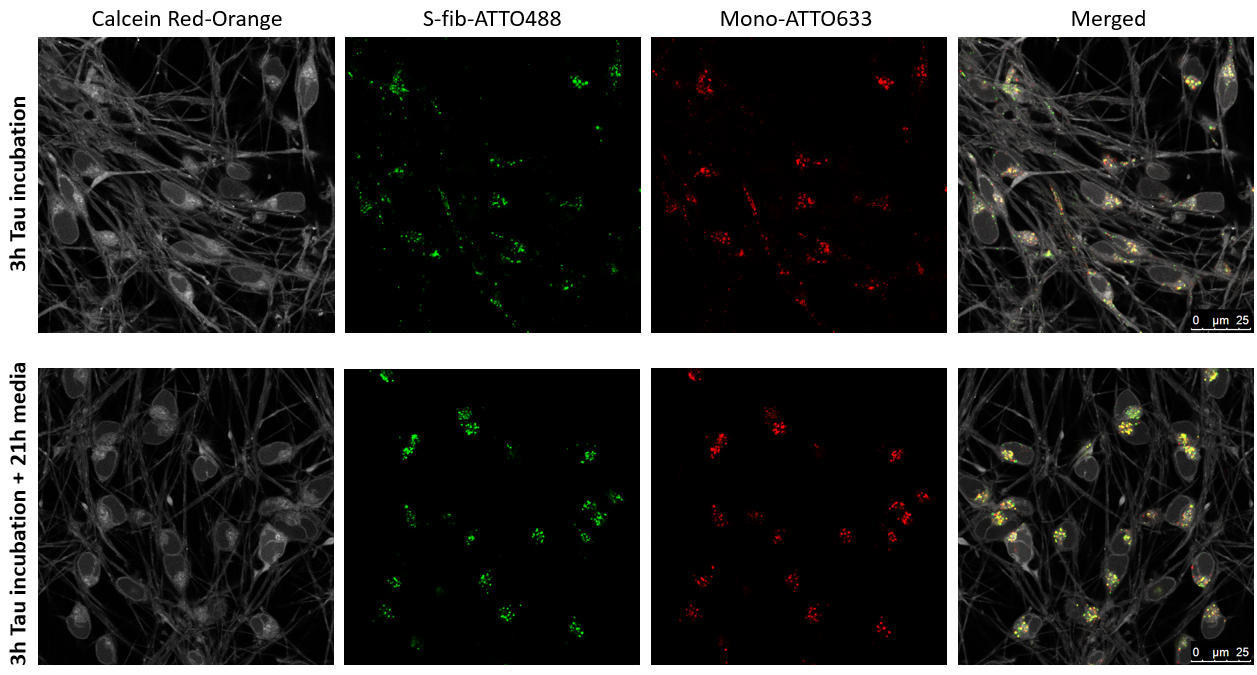


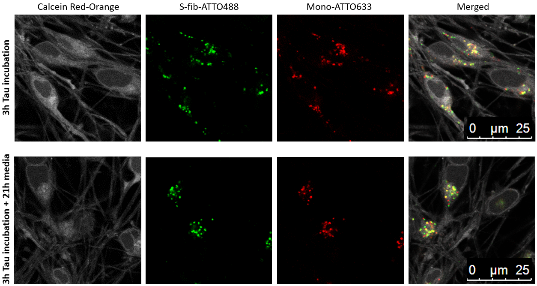


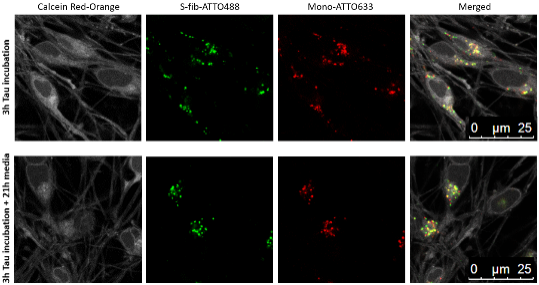


**B**

**A**


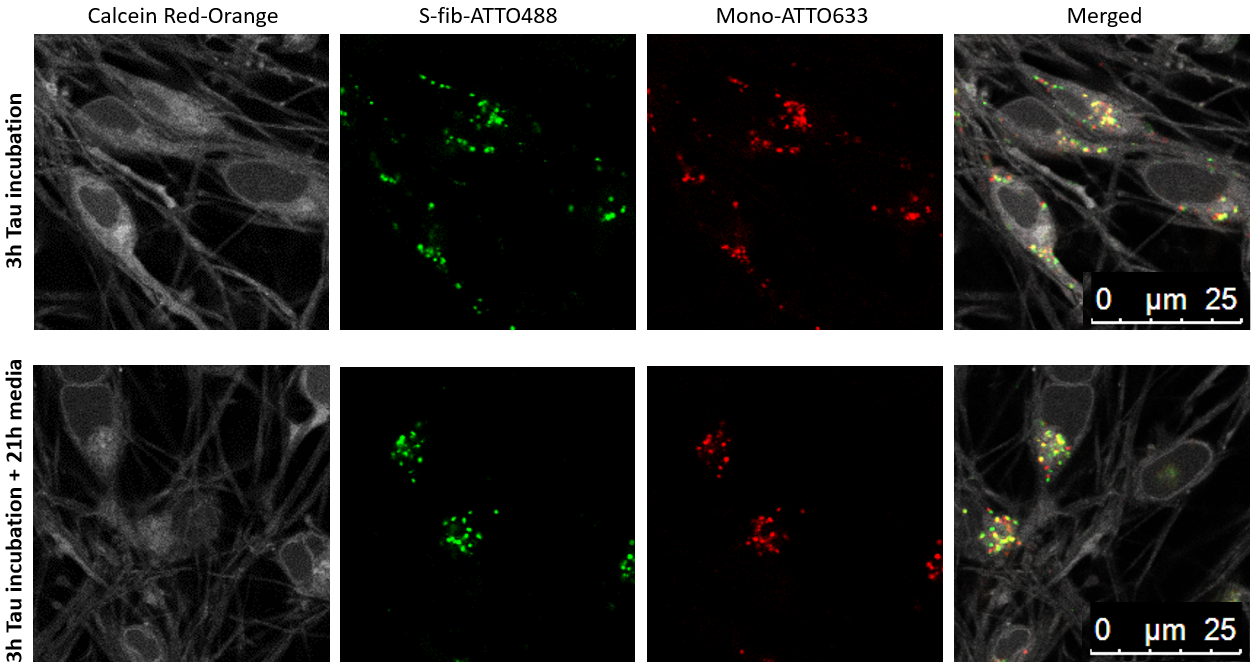


**Supplementary Figure S15** Confocal microscopy of cellular localization of Tau monomers and small fibrils after short and long incubation in LUHMES neurons. **A** Confocal imaging of LUHMES neurons co-treated with 500 nM Tau monomers labeled with ATTO633 (Mono-ATTO633) and 100 nM small fibrils labeled with ATTO488 (S-fib-ATTO488). Cells were incubated with Tau for 3 hours, and imaging was performed immediately or after 21 hours of incubation in the standard differentiation media without Tau. Calcein red-orange was used as a cell tracker. **B** Higher magnification of the square in part A.
